# Is DNA methylation in the brain a mechanism of alcohol use disorder?

**DOI:** 10.3389/fnbeh.2023.957203

**Published:** 2023-01-26

**Authors:** Justyna Jarczak, Michalina Miszczak, Kasia Radwanska

**Affiliations:** Laboratory of Molecular Basis of Behavior, Nencki Institute of Experimental Biology, Polish Academy of Sciences, Warsaw, Poland

**Keywords:** DNA methylation, cytosine modifications, epigenetic modifications, animal models, alcohol use disorder

## Abstract

Alcohol use disorder (AUD) is a worldwide problem. Unfortunately, the molecular mechanisms of alcohol misuse are still poorly understood, therefore successful therapeutic approaches are limited. Accumulating data indicate that the tendency for compulsive alcohol use is inherited, suggesting a genetic background as an important factor. However, the probability to develop AUD is also affected by life experience and environmental factors. Therefore, the epigenetic modifications that are altered over lifetime likely contribute to increased risk of alcohol misuse. Here, we review the literature looking for the link between DNA methylation in the brain, a common epigenetic modification, and AUD-related behaviors in humans, mice and rats. We sum up the main findings, identify the existing gaps in our knowledge and indicate future directions of the research.

## 1. Introduction

Alcohol use disorder (AUD) is a medical condition characterized by an impaired ability to stop or control alcohol use despite adverse social, occupational, or health consequences (American Psychiatric Association, 2013). According to the World Health Organization (WHO) every year 3 million deaths world-wide result from harmful use of alcohol (Glantz et al., 2020). Thus, alcohol misuse is the leading worldwide problem which seriously affects not only addicted individuals but also their families and society (Rehm and Shield, 2019). Unfortunately, the complex etiology of AUD is still not fully understood and, therefore, the therapeutic choices are very limited (Nielsen et al., 2012; Egervari et al., 2021).

There are multiple factors that contribute to the etiology of AUD, including: genetic and epigenetic variability, social and cultural factors as well as adverse life experiences (Tawa et al., 2016; Berkel and Pandey, 2017; Carvalho et al., 2019; Nestler and Lüscher, 2019; Nalberczak-Skóra et al., 2020; Egervari et al., 2021; Gelernter and Polimanti, 2021; Kirsch and Lippard, 2022). Based on the meta-analysis of twin studies, the heritability of AUDs is estimated to be around 50% (Verhulst et al., 2015). For example, the offspring and siblings of AUD patients are five times more likely to misuse alcohol as compared to the controls (Midanik, 1983; Bierut et al., 1998). The genetic basis of addiction is confirmed by genome-wide association studies (GWAS) which indicated several gene variants correlating with the risk of AUD (Gelernter and Polimanti, 2021). *Alcohol dehydrogenase 1B* (*ADH1B*) and *aldehyde dehydrogenase 2* (*ALDH2*), the genes that take part in ethanol metabolism, were the most frequently validated with some functional variants associated with the resilience or susceptibility to AUD (Kranzler et al., 2019; Egervari et al., 2021; Gelernter and Polimanti, 2021). Importantly, *ADH1B and ALDH2* alleles explain a very small fraction of overall phenotypic variance, which is accounted for not only by polygenicity of AUD (contribution of thousands of alleles with small effects), but also by the contribution of epigenetic and environmental factors. In order to provide a successful therapy of AUD, the brain mechanism affected by all these factors must be understood.

Multiple epigenetic mechanisms have been implicated in AUD etiology, including histone modifications, expression of non-coding RNAs (reviewed elsewhere, Ponomarev, 2013; Berkel and Pandey, 2017; Bourguet et al., 2018; Gowen et al., 2021; Longley et al., 2021; Rodriguez, 2021; Zhu et al., 2022) and DNA methylation. In this review we focus on the literature that concerns DNA methylation in the brain as a plausible, epigenetic mechanism of AUD development. DNA methylation plays a crucial role in tissue-specific gene expression, cellular differentiation, X chromosome inactivation, imprinting of parental alleles, and repetitive element silencing (Kohli and Zhang, 2013). However, there is also a growing body of evidence indicating that the methylation of DNA is not only inherited and established during development, but it also constantly changes throughout individual’s lifetime likely affecting physiological and pathological neuronal processes (Sharma, 2015; Szyf, 2015; Bierer et al., 2020; Daskalakis et al., 2021). It was shown that DNA methylation and demethylation are important in fully differentiated cells (Nabel et al., 2012; Traube and Carell, 2017). As an example, locus-specific DNA demethylation and *de novo* methylation are induced by neuronal activation arguing that DNA methylation is important for normal brain function (Traube and Carell, 2017). The active demethylation at the gene promoter is a trigger for neural plasticity (Nabel et al., 2012). DNA methylation and demethylation influence memory processes (Tognini et al., 2015). DNA methylation is also affected by internal body states (e.g., stress and aging) and environmental conditions (e.g., drugs and pollution) (Rustad et al., 2019). Furthermore, the abnormalities in DNA methylation were observed in several diseases (Rasmussen and Helin, 2016; Hwang et al., 2017; Wojdacz et al., 2019). For example, many cancer types are characterized by decreased global methylation levels, except for promoter regions of crucial regulatory and tumor suppressor genes which are hypermethylated and their expression is therefore silenced (Traube and Carell, 2017). Moreover, several neuroepigenetic changes have been recently described in neurodevelopmental, psychiatric and neurodegenerative disorders (Cholewa-Waclaw et al., 2016), such as: autism (Aspra et al., 2022), schizophrenia (Song et al., 2022), posttraumatic stress disorder (Montalvo-Ortiz et al., 2022), heroin addiction (Kozlenkov et al., 2017), and Parkinson’s disease (Kaut et al., 2022).

**FIGURE 1:**
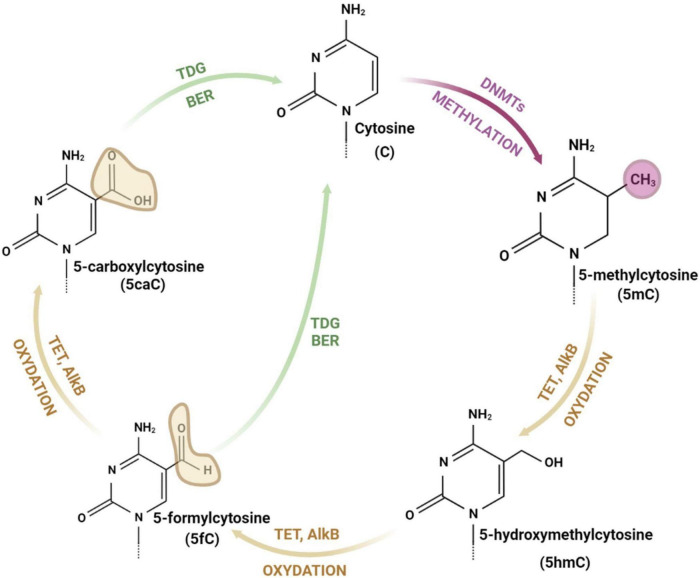
The cycle of cytosine modifications. The cytosine is methylated by DNA methyltransferases (DNMTs) and demethylated through iterative stages of cytosine oxidation (with 5-hydroxymethylcytosine, 5-formylcytosine and 5-carboxylcytosine as intermediate forms) catalyzed by ten-eleven translocation enzymes (TETs), alpha-ketoglutarate dependent dioxygenases (AlkB), thymine DNA glycosylase (TDG), and base excision repair (BER) pathway. drawings are made in Bio Render.

**FIGURE 2:**
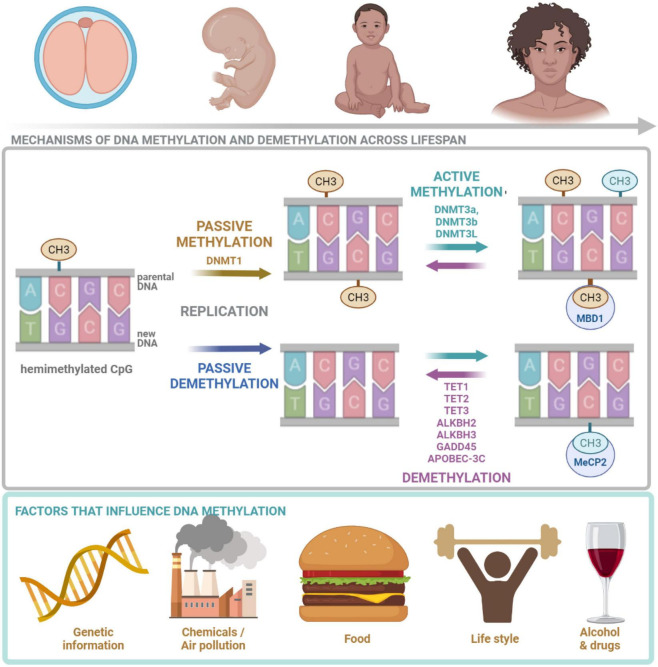
DNA methylation across lifespan. DNA methylation is crucial at all stages of human life. It is regulated by many factors including: genetic landscape, air pollution, diet, lifestyle as well as drugs and alcohol. DNMT1, responsible for maintenance methylation, acts on the hemimethylated CpG site generated by the replication. DNMT3a and DNMT3b, responsible for *de novo* methylation, attach methyl groups in unmethylated CpG sites. DNA methylation may be passive, defined as the failure of maintenance methylation after DNA replication or active, independent to replication, carried out by several enzymes. Demethylation process is carried out and controlled by TETs. MeCP2 is able to bind specifically to methylated DNA and repress transcription from methylated gene promoter. CH3, methyl group; CpG, cytosine/guanine site; DNMT1, DNA methyltransferase 1; DNMT3a, DNA methyltransferase 3a; DNMT3b, DNA methyltransferase 3b; DNMT3, DNA methyltransferase 3L; TET1, TET methylcytosine dioxygenase 1; TET2, methylcytosine dioxygenase 2; TET3, methylcytosine dioxygenase 3; ALKBH2, alpha-ketoglutarate-dependent dioxygenase AlkB homolog 2; ALKBH3, alpha-ketoglutarate-dependent dioxygenase AlkB homolog 3; GADD45, growth arrest and DNA damage inducible protein 45; APOBEC-3C, apolipoprotein B mRNA-editing enzyme complex; MeCP2, Methyl-CpG binding protein 2. The figure is made in Bio Render.

In the context of alcohol misuse, not only alcohol consumption, but also life experiences that affect propensity for AUD, such as early-life (Vrettou et al., 2017; Bendre et al., 2019) and prenatal stress (Dong et al., 2018), voluntary alcohol consumption (Bendre et al., 2019) as well as adolescent alcohol exposure (Asimes et al., 2017; Sakharkar et al., 2019) affect DNA methylation. Here we review the literature with the aim to look for the link between the changes of DNA methylation status in the brain and development of AUD. Beginning with the biochemical basis of DNA methylation, next we will describe how alcohol exposure affects activity and expression of the enzymes responsible for cytosine modifications (Figures 1, 2) as well as DNA methylation status (Supplementary Table 1). We will also review the literature that considers DNA methylation as a mechanism that links alcohol use, adverse life experiences and AUD-related behaviors. The review includes human studies as well as those conducted using animal models. As addiction-related behaviors are primarily driven by the brain reward system (See, 2002; Gipson et al., 2013; Keistler et al., 2017; Stefaniuk et al., 2017; Beroun et al., 2018; Namba et al., 2018) we will focus on DNA methylation in the brain. We included papers published up to 31st of December 2022. The key terms used were: DNA methylation, brain, alcohol and AUD.

## 2. Biochemistry and functional consequences of DNA (de)methylation

The DNA is composed of a chain of complementary base pairs linked by hydrogen bonds and hydrophobic interactions between adenine and thymine (A-T), and cytosine and guanine (C-G). The DNA structure is conservative and only a few bases can be modified (Traube and Carell, 2017). Cytosine consists of a heterocyclic aromatic ring with an amino group attached at 4th position and a keto group attached at 2nd position (Figure 1). The 5th position (C5) of the cytosine ring is uniquely located to attach a methyl group and tolerate other modifications without destabilizing DNA structure and base pairing (Hardwick et al., 2018).

Addition of a methyl group to the cytosine base at C5 results in generation of 5-methylcytosine (5mC) (Figure 1). This reaction is catalyzed by DNA methyltransferases (DNMTs). The mammalian genome cytosines are methylated mostly in the cytosine and guanine dinucleotides (CpG) and in the native DNA approximately 70% of CpG islands (DNA fragments with frequent CpGs) are methylated (Hardwick et al., 2018). Importantly, DNA methylation is a fully reversible process, which occurs through direct restoration of the original nucleobase or indirectly by replacement of the methylated nucleobase with an unmodified nucleobase (Shen et al., 2014; Xu and Bochtler, 2020). Other forms of modified cytosines are related to the demethylation process and appear as a result of 5mC oxidation and subsequent stages of the DNA repair pathway (Ross and Bogdanovic, 2019). 5mC demethylation relies on the oxidation and is performed by α-KG/Fe(II)-dependent proteins: Ten-eleven translocation (TET) enzymes (Chen and Riggs, 2011) and alpha-ketoglutarate-dependent dioxygenases (AlkB) (Bian et al., 2019; Perry et al., 2021). 5-hydroxymethylcytosine (5hmC) is an intermediate form and modification of cytosine. 5hmC is mostly present in the promoter region of actively expressed genes, which proves its role in the acceleration of transcription (Traube and Carell, 2017). TET enzymes (TET1, TET2, and TET3), conduct the iterative oxidation resulting in two more forms of cytosine: 5-formylcytosine (5fC) and 5-carboxylcytosine (5caC) (Chen and Riggs, 2011; Bian et al., 2019; Perry et al., 2021). Both modifications can be removed by thymine DNA glycosylase (TDG) and repaired by the base excision repair (BER) pathway (Ross and Bogdanovic, 2019). AlkB demethylases are enzymes, which are able to directly remove a methyl group from the nucleic acid bases (Perry et al., 2021). They contain, similarly to TET enzymes, the highly conserved N-terminal -hairpin-like element for DNAbase recognition and the C-terminal catalytic domain and probably play a crucial role in epigenetic modulation (Bian et al., 2019). AlkB proteins identified in *Escherichia coli* are protective factors against the cytotoxicity of methylating agents and repair the DNA lesions generated in single-stranded DNA. Human and mice AlkB homologs (AlkBH2 and AlkBH3) catalyze the methyl group removal from 1-methyladenine and 3-methylcytosine (Xu and Bochtler, 2020).

The influence of DNA methylation on gene expression can be direct, e.g., by preventing transcription factors binding. It can also result in the changes of chromatin conformation. Moreover, there are several proteins that bind to 5mC, modulate chromatin architecture and influence binding of transcription machinery, such as methyl CpG binding protein 2 (MeCP2) and methyl-CpG binding domain protein 1 (Mbd1) (Hardwick et al., 2018).

Generally, the presence of methylated CpG islands in the genome is linked with repressed (inactive) chromatin state (Hardwick et al., 2018). Moreover, DNA methylation at gene promoters is associated with repression, while methylation in gene bodies is associated with active transcription (Brenet et al., 2011). However, in the neurons from the human PFC the levels of 5mC both in the promoters and gene bodies negatively correlate with gene expression (Hardwick et al., 2018). 5hmC is concentrated in gene bodies and associated with active gene transcription. These findings indicate that gene expression is modulated by the DNA methylation status (conversion from 5mC to 5hmC and inversely) in the promoters and gene bodies.

## 3. Enzymes and their role in alcohol-related behaviors

### 3.1. DNA methyltransferases (DNMTs)

DNMTs add the methyl group to the cytosine base (Traube and Carell, 2017). Considering the molecular mechanism of cytosine methylation two types of processes can be discerned: active and passive. Passive methylation is executed by DNMT1 which favors hemimethylated CpGs (Hardwick et al., 2018) and is regulated by 5hmCs, as they reduce DNMT1 activity (Rasmussen and Helin, 2016). DNMT1 methylates cytosines after DNA replication at the newly synthesized DNA strand, if the cytosine on the template strand is methylated. The process takes place mostly during development and cell division. It transfers the parental methylation pattern during reproduction and makes it inheritable (Traube and Carell, 2017; Figure 2). Therefore, the passive methylation is also referred to as the maintenance methylation (Hardwick et al., 2018). Active *de novo* methylation of CpG sites repeatedly occurs during an individual’s life and is held by DNMT3a and DNMT3b (Hardwick et al., 2018). DNMT3L does not directly catalyze the methylation (it lacks the methyltransferase motif), however, it stimulates the activity of DNMT3a and DNMT3b (Chen and Riggs, 2011). There is an interaction between *de novo* DNMTs and maintenance DNMT1—*de novo* methylation in somatic cells conducted by DNMT3a and DNMT3b restores methylation of CpGs missed by DNMT1 during replication (Chen and Riggs, 2011). Both passive and active DNA methylations are affected by multiple factors including: molecular landscape of the cell, environmental pollution, diet and life-style as well as alcohol and drugs (Figure 2). It was found that global dysfunction of the activity of only one of DNMTs can be lethal as well as may lead to many cellular abnormalities. In the case of DNMT1 and 3b, lethality was observed in early embryogenesis, while in the case of DNMT3a it was postnatal (Traube and Carell, 2017).

No differences in the total DNMTs protein levels were observed in mPFC of the rats exposed to ethanol vapor for 14 weeks, as compared to alcohol-naive animals. However, the levels of DNMT1 were significantly increased in neurons of the alcohol group. Furthermore, increased levels of DNMT1 were associated with increased levels of DNA methylation (Barbier et al., 2015). Interestingly, infusion of DNMTs inhibitor, N-Phthalyl-L-tryptophan (RG108), directly into mPFC prevented the escalation of alcohol self-administration in alcohol-exposed rats, but not in the control rats. It is worth adding that infusion of RG108 did not influence locomotor activity of rats, supporting a selective role of RG108 in escalation of alcohol consumption. The role of DNMTs in PFC in alcohol consumption and preference was also confirmed in the rat model using 5′AZA, a potent DNMT1 inhibitor (Qiao et al., 2017). After 5′AZA infusion, alcohol intake was decreased, as compared to the rats injected with DMSO.

In the study of Sakharkar et al. (2019) the effect of adolescent alcohol exposure on the DNMTs and growth arrest and damage inducible proteins 45 (GADD45s) expression was studied in the rat amygdala. The increased DNMTs activity was observed in the alcohol-exposed adolescent group 24 h after ethanol injection. Considering *DNMTs* expression, the significant difference was observed only in the case of *DNMT3b*, which expression was decreased 1 h after ethanol injection. In adult rats, similarly to adolescent ones, the increased DNMTs activity was observed in the alcohol-exposed group, and this change was accompanied by increased expression of *DNMT3b* and *DNMT1*. Increased activity and expression of DNMTs in adolescent and adult rats after alcohol exposure may suggest an increase in methylation processes induced by alcohol. Also the upregulated expression of *DNMT1* was observed in NAc of the mice consuming alcohol, as compared with the animals consuming only water (Warnault et al., 2013). Auta et al. (2017), studying the cerebellum of the rats fed on an ethanol diet for 15–16 days, found no differences in the expression of *DNMT1, DNMT3a* and *DNMT3b* in the alcohol-fed animals as compared to rats during ethanol withdrawal (24 h) or animals fed with a standard diet. Similarly, in the cerebellum samples from AUD patients, no differences in the expression of *DNMT1, DNMT3a, DNMT3b* were detected (Gatta et al., 2017). However, the methylation index [defined as the S-adenosyl methionine (SAM) and S-adenosylhomocysteine (SAH) ratio] was increased in the rats fed with an alcohol diet. The conversion of SAM into SAH and thus SAM/SAH ratio are a reflection of alterations in DNMTs activity, that translates into the changes in DNA methylation levels (Auta et al., 2017).

DNMTs inhibitors reduced alcohol consumption in most of the experimental paradigms. Inhibition of DNMTs by intracerebroventricular RG108 infusion resulted in decreased alcohol intake (Barbier et al., 2015). Systemic injection of 5′AZA decreased alcohol consumption in the rats pre-exposed to alcohol, but not in alcohol-naive animals (Sakharkar et al., 2019). Systemic administration of 5′AZA also reduced excessive alcohol intake in mice (Warnault et al., 2013). The reduction was observed in different conditions: in binge alcohol consumption, which significantly decreased during the first 4 h of alcohol access; and in 24-h alcohol-drinking session. The effect of 5′AZA was specific to alcohol and it did not modify water and saccharose consumption (Warnault et al., 2013). These findings contradict, however, one study where the authors observed that intracerebroventricular injections of 5′AZA increased alcohol consumption (Qiang et al., 2014). This effect was reversed by SAM administration (cofactor in the methylation reaction and donor of the methyl group) (Qiang et al., 2014).

Furthermore, in human studies, Guidotti et al. (2013) investigated the processes of DNA methylation and DNA demethylation in the PFC of psychotic patients with a history of alcohol misuse. Although the psychotic patients showed increased expression of *DNMT1*, as compared to non-psychotic controls, no effect of alcohol history on *DNMT1* levels was found (Guidotti et al., 2013).

Conclusions: In most of the studies conducted so far voluntary ethanol consumption did not change the total DNMTs levels in the brain. Significant differences in *DNMT1* expression were observed between the ethanol-exposed and ethanol-naive animals only when ethanol was injected or inhaled (Sakharkar et al., 2019), or when authors focused on cell type-specific expression (Barbier et al., 2015). Despite the lack of the common effects of alcohol consumption on DNMTs expression, the changes in DNA methylation were often observed. Moreover, inhibition of DNMTs (systemic, intracerebroventricular or local in PFC) consistently reduced alcohol consumption and preference, supporting the significant role of DNA methylation in the regulation of alcohol drinking.

### 3.2. DNA demethylation enzymes

DNA can be demethylated in a passive or active process. Passive demethylation occurs due to the omission of methylation after replication (Rasmussen and Helin, 2016), while active demethylation requires the activity of AlkB or/and TET enzymes (Chen and Riggs, 2011; Figure 2). There are three mammalian TET enzymes identified so far: TET1, TET2, and TET3 (Chen and Riggs, 2011). Overexpression of TET1 reduces the frequency of 5mCs in the genome (Kohli and Zhang, 2013). The role of TET enzymes have been described in relation to neurogenesis and cognition (Rudenko et al., 2013; Zhang et al., 2013; Ross and Bogdanovic, 2019). Knockout of TET1 decreases 5hmC levels in the mouse brain and results in down-regulation of synaptic plasticity-related genes: *neuronal PAS domain protein 4 (Npas4), fos proto-oncogene (c-Fos), early growth response 2 (Egr2)*, and *early growth response 4 (Egr4*), increased hippocampal long-term depression and impaired memory (Rudenko et al., 2013). The knockout of *TET1* in mice does not influence locomotion, anxiety and depression-related behaviors or fear memory formation, however, it causes the impairment of fear memory extinction, which was connected with the hypermethylation of promoter region and down-regulation of Npas4 and c-Fos (Rudenko et al., 2013). The knockout of *TET1* was also shown to result in defective adult neurogenesis, impairment in the maintenance of the neural progenitor cells, the expression of several genes related to synaptic plasticity: *galanin and GMAP prepropeptide (Gal), chondroitin sulfate proteoglycan 4 (Ng2), neuroglobin (Ngb), potassium channel tetramerization domain containing 14 (Kctd14)*, and *ATP synthase peripheral stalk subunit D (Atp5h)* as well as spatial memory deficits (Zhang et al., 2013).

The data related to TETs function and alcohol-related behaviors are very scarce so far. No changes in *TET1, TET2*, and *TET2* expression were observed in the PFC and NAc of the high vs. low drinking mice sacrificed 10 days after alcohol withdrawal (Repunte-Canonigo et al., 2014). Guidotti and collaborators studied the processes of DNA demethylation in psychotic patients with a history of alcohol misuse (Guidotti et al., 2013). The authors investigated whether the alcohol misuse alters the expression of the genes from DNA demethylation network in the PFC. They reported an increase of *TET1* mRNA expression, as well as a decrease of *APOBEC-3C* [apolipoprotein B mRNA-editing enzyme complex involved in turning 5hmC into 5-hydroxylmethyluracil (5hmU)] mRNA expression in the psychotic patients with AUD, as compared to the patients without misuse history. Up to date the role of AlkB enzymes in AUD-related behaviors is unknown.

Conclusions: There are still only a few studies which directly investigate the enzymes from the DNA demethylation pathway in the context of alcohol use. Thus, to draw any conclusion about the role of these enzymes in AUD-related behaviors, further research is needed. In particular, it would be of great interest to validate whether AlkB proteins affect DNA demethylation in the mammalian brain exposed to ethanol.

### 3.3. Thymine DNA glycosylase (TDG) and alcohol-related behaviors

Thymine DNA glycosylase (TDG) is a key DNA repair enzyme (Nabel et al., 2012). Its crucial role for animal survival was confirmed in the mouse model, as TDG knockout mice are lethal at the embryonic stage, in contrast to UDG (uracil DNA glycosylase) knockout mice which are viable, however, sterile. TDG is probably the only DNA glycosylase inducing such a phenotype (Nabel et al., 2012). It removes the oxidized cytosine intermediates: 5fC and 5caC, which allows for restoration of unmethylated cytosine by base excision repair (BER) pathway (Ross and Bogdanovic, 2019; Figure 2). This repair mechanism, with TDG as a base excision catalase and components of BER pathway [enzymes: Poly (ADP-ribose) polymerase 1 (PARP1), AP endonuclease (APE1), X-ray repair cross complementing 1 (XRCC1)], is a main route of active DNA demethylation (Rasmussen and Helin, 2016; Traube and Carell, 2017). The binding and excising mismatched pyrimidines (base pairs: G:U and G:T) are the major activities of TDG. TDG recognizes 5fC and 5caC and removes the oxidized cytosine. The basic site is then repaired within the BER pathway, resulting in the restoration of the unmodified cytosine state. The lack of TDG in embryonic stem cells caused a significant increase, while overexpression of TDG led to the decreased levels of both oxidized forms of cytosine (Rasmussen and Helin, 2016). Furthermore, *in vitro* studies confirmed the high activity of TDG to excise 5fC and 5caC, but not 5mC and 5hmC, which emphasizes the need to modify the cytosine to the carbonyl and formyl forms as crucial steps in the active demethylation process (Traube and Carell, 2017). In the studies of zygotes, it was shown that inhibition of BER elements significantly increased the levels of DNA methylation, which proves the crucial role of the BER pathway in active demethylation (Chen and Riggs, 2011). As far as we know there are no papers to date that investigated the role of TDG and BER pathway in alcohol-related behaviors.

Gadd45 proteins contribute to the DNA demethylation by recruiting TDGs to genomic loci (Tulisiak et al., 2017). *Gadd45b* expression is increased in the NAc of the mice injected with alcohol and this correlates with higher 5mC and 5mhC within *Bdnf9a* promoter as well as decreased *Bdnf9a* mRNA levels (Gavin et al., 2016). The expression of *Gadd45a, Gadd45b, Gadd45g* is also increased in the amygdala of the adolescent rats exposed to alcohol, as compared to the alcohol-naive controls. In the adult rats, the expression of *Gadd45g* was significantly decreased in the amygdala of the alcohol-exposed adult rats (Sakharkar et al., 2019). Thus, although still very scarce, the data suggest that alcohol exposure induces DNA repair processes.

### 3.4. Methyl CpG binding proteins (MeCP2)

MeCP2 binds tightly to 5mC (Figure 2) and plays an important role in the alternation of the chromatin structure and regulation of transcription (Schmidt et al., 2020; Bin Akhtar et al., 2022). MeCP2 is also involved in modulation of RNA splicing (Liyanage et al., 2017). MeCP2 mutation was originally discovered to be associated with Rett syndrome (Amir et al., 1999; Wan et al., 1999), however, now it is linked with a plethora of neurologic and psychiatric disorders, including cocaine addiction (Ausió, 2016).

The binge-like and continuous ethanol exposure of the differentiating embryonic brain-derived neural stem cells upregulated, while alcohol withdrawal decreased, MeCP2 expression. MeCP2 upregulation was associated with increased levels of 5hmC and decreased levels of 5mC. Inversely, MeCP2 downregulation during withdrawal was associated with decreased levels of 5hmC and increased levels of 5mC (Liyanage et al., 2017). Despite the study being conducted *in vitro*, it is an excellent example of an ethanol-induced epigenetic mechanism of gene expression regulation. In the study of Sakharkar and collaborators, significantly higher levels of MeCP2 at the Npy promoter was observed in the amygdala of adult rats exposed to alcohol in adolescence (Sakharkar et al., 2019). Moreover, MeCP2 expression was significantly increased both in mPFC and NAc of mice with history of alcohol misuse (Repunte-Canonigo et al., 2014).

MeCP2308/Y mice (with a truncation of MeCP2 at amino acid 308 resulting in the loss of the C-terminal region of the protein) are more sensitive than their wild-type counterparts both to the psychostimulant effect of a moderate dose of ethanol as well as to the intoxicating effects of a higher dose of ethanol. Additionally, while MeCP2308/Y mice did not differ from wild-type mice in ethanol preference in a 24 h 2 bottle choice test, they drank significantly less in a 2 h limited access paradigm, and did not increase their ethanol intake after intermittent exposure to ethanol vapors as did wild-type mice (Repunte-Canonigo et al., 2014). These results suggest that MeCP2-regulated genes modulate ethanol sensitivity and intake.

## 4. CpG methylation and alcohol use

Long-term molecular changes induced in the brain by alcohol use are believed to drive behaviors related to AUD (Nestler and Lüscher, 2019). Accumulating data indicate that epigenetic processes, such as DNA methylation, are induced by alcohol (De Sa Nogueira et al., 2019). Here, we focus on the genes that are differentially methylated in the brain reward system of the animals drinking alcohol and AUD patients. The differentially methylated genes are listed in Supplementary Table 1.

### 4.1. Animal studies

Epigenetic studies in animal models allow for the analyses of differentially methylated CpGs in well controlled experimental conditions. Few such analyses were conducted so far with the use of rhesus monkeys (Cervera-Juanes et al., 2017), but generally mice and rats are considered as the simplest animal models for studying alcohol-related behaviors (Goltseker et al., 2019).

Significant differences in the methylation pattern were observed in the NAc of the rhesus monkeys that escalated alcohol consumption for 12 months, as compared to low drinkers (Cervera-Juanes et al., 2017). Using epigenome-wide association studies (EWAS), the authors identified 17 differentially methylated regions (DMRs), including 14 with methylation levels that were correlated with average daily alcohol consumption. The size of the DMRs ranged from 29 to 158 bp (mean = 63.7), including 4–19 CpGs per DMR (mean = 8.06). Eight of the DMRs mapped to genes implicated in modulation of synaptic plasticity, divided into presynaptic [*kirre-like nephrin family adhesion molecule 3 (Kirrel3), low-density lipoprotein receptor-related protein 5 (Lrp5)* and *neurotrimin (Ntm)]* and postsynaptic [*rho guanine nucleotide exchange factor 7 (Arhgef7), Cadherin 5 (Cdh5), G protein-coupled receptor 39 (Gpr39), janus kinase and microtubule interacting protein 1 (Jakmip1)*, and *neurobeachin (Nbea)*]. In the study of the alcohol preferring rats, global increase in the 5hmC and 5mC levels were observed in NAc of the animals drinking alcohol, as compared to alcohol-naive animals. From the selected alcohol addiction-related genes, *prodynorphin (Pdyn)* had increased levels of mRNA, and decreased levels of 5mC in the promoter region. While the levels of *opioid receptor kappa 1 (Oprk1)* mRNA were increased, the changes in 5mC levels within this gene were not observed. Interestingly, the levels of 5hmC in the *Oprk1* promoter were significantly increased. The discrepant results suggest that DNA methylation is not the only mechanism involved in the regulation of ethanol-affected genes (Niinep et al., 2021). Maier et al. (2020) investigated glial cell line-derived neurotrophic factor (*Gdnf*) mRNA expression and promoter methylation in the NAc and Ventral Tegmental Area (VTA) of alcohol-drinking rats 24-h after alcohol consumption and during withdrawal. *Gdnf* expression during alcohol consumption and withdrawal correlated with DNA methylation of the promoter and negative regulatory element (NRE) of *Gdnf* gene (Maier et al., 2020). Since infusion of Gdnf into VTA reduces alcohol intake, reward as well as relapse after withdrawal (Maier et al., 2020), *Gdnf* DNA methylation in the NAc and VTA may control alcohol-related behaviors.

Cui et al. (2020) investigated DNA methylation in mPFC of alcohol-exposed rats. With the use of reduced representation bisulfite sequencing, the authors detected methylation levels and then verified mRNA expression of several genes. The methylation levels in the promoter regions of *neurotrophin 3* (*Ntf3*) and *protein phosphatase magnesium-dependent 1 gamma* (*Pgm1G*) were increased, while mRNA levels of those genes decreased in alcohol-exposed rats as compared to the alcohol-naive controls. Consistent results were also observed for *huntingtin associated protein 1* (*Hap1*) and *dual specificity phosphatase 1 (Dusp1)* with decreased methylation in the promoter and increased mRNA levels in alcohol-exposed groups (Cui et al., 2020). Qiao et al. (2017) analyzed two target genes in mPFC of the rats drinking alcohol: *Ntf3* and its *receptor neurotrophic receptor tyrosine kinase 3* (*Ntrk3*). There were no differences in *Ntrk3* expression after alcohol consumption but its expression significantly decreased after treatment with an inhibitor of methylation, 5′AZA. *Ntf3* was downregulated after alcohol consumption, with reversed expression after 5′AZA treatment. This finding was confirmed by Cui et al. (2020).

Qiang et al. (2014) analyzed methylation of the glutamate ionotropic receptor *NMDA type subunit 2B (Grin2B)* in mPFC of chronic intermittent ethanol (CIE)-exposed mice, as compared to air-exposed animals. This gene was selected on the basis of the previous results, indicating the upregulation of *Grin2B* expression (Qiang et al., 2007) and DNA demethylation of *Grin2B* promoter in neuronal cultures after CIE exposure (Qiang et al., 2010). Significant demethylation of 18 CpGs of *Grin2B* promoter, as well as increased expression of *Grin2B* mRNA, was observed in CIE-exposed mice. The involvement of ethanol consumption in the regulating of epigenetic modifications was additionally confirmed with the use of quantitative chromatin immunoprecipitation assay (qCHIP). Mice treated with ethanol had increased levels of H3K9 acetylation in *Grin2B* promoter regions (Qiang et al., 2014).

Barbier et al. (2015) found seven genes coding for synaptic plasticity proteins to have significantly decreased expression in mPFC of alcohol-dependent rats 3 weeks after chronic alcohol exposure, compared to the alcohol-naive controls. The list of the genes included: *synaptotagmin 1 (Syt1), synaptotagmin 2 (Syt2), calcium voltage-gated channel subunit alpha1 A (Cacna1a), calcium voltage-gated channel subunit alpha1 I (Cacna1i), WNK lysine deficient protein kinase 1 (Wnk1), WNK lysine deficient protein kinase 2 (Wnk2), potassium voltage-gated channel subfamily C member 1 (Kcnc1)*. To investigate if DNA methylation is a mechanism that regulates their transcription, an inhibitor of DNA methylation, RG108, was used. RG108 treatment prevented the downregulation of *Syt1, Syt2, Cacna1a*, and *Wnk2*. Moreover, RG108 treatment prevented alcohol-induced hypermethylation on the first exon of *Syt2* and no significant differences were observed in DNA methylation levels on the promoter region of *Cacna1a*. Thus, alcohol consumption decreases expression of *Syt2* by increased DNA methylation on its exon. Interestingly, *Syt2* knockdown in PFC had no effect on alcohol consumption during free access but modified compulsive-like alcohol drinking (upon adulteration with quinine) (Barbier et al., 2015).

In the rat amygdala decreased DNA methylation and increased mRNA expression was observed after single alcohol injection for *hypoxia-inducible factor 3, alpha subunit* (*Hif3a)* and *solute carrier family 10 member 6* (*Slc10a6*) (Krishnan et al., 2022). Interestingly, knockdown of *Hif3a* expression in the central nucleus of amygdala (CeA) attenuated acute ethanol-induced anxiolysis. Decreased *activity regulated cytoskeleton associated protein* (*Arc*) gene methylation and increased mRNA expression was also found in the amygdala of mice drinking alcohol (Pagano et al., 2022). *Arc* gene methylation levels correlated with alcohol seeking during withdrawal, not alcohol consumption; while *Arc* mRNA levels were increased in mice diagnosed with AUD-resistant phenotype. The authors further showed that knockdown of Arc in CeA regulates alcohol motivation and seeking during relapse induced by alcohol-predicting cues, but it has no effect on alcohol consumption. Finally, Arc gene methylation in blood samples was correlated with alcohol consumption frequency and size of the amygdala in the IMAGEN cohort (Schumann et al., 2010).

The role of adolescent (28–41 PND) alcohol exposure on DNA methylation in adulthood (92 PND) was investigated in the rat amygdala (Sakharkar et al., 2019). The authors selected *brain derived neurotrophic factor* (*Bdnf*) exon IV and *neuropeptide Y* (*Npy*) promoters for DNA methylation analysis. Both genes affect anxiety states, which may contribute to AUD etiology (Palmisano and Pandey, 2017). Significantly higher levels of DNA methylation in both regions were observed in alcohol-exposed adult rats as compared to the alcohol-naive controls. The lack of mRNA expression analysis limits conclusions of this study. However, these results indicate that adolescent alcohol exposure is an important factor influencing DNA methylation status in the adult brain. Moreover, in the NAc of alcohol-exposed male rats the methylation of *Bdnf* promoter was significantly reduced while in mPFC significantly increased (Nieto et al., 2022), indicating that alcohol consumption differentially affects *Bdnf* methylation in different brain regions.

In the studies of the rat cerebellum, authors found that methylation index (defined as the SAM/SAH ratio) was increased, while SAH level decreased in the group of ethanol exposed rats. Thus increased methylation index in the cerebellum of alcohol exposed rats, suggests DNA hypermethylation. These parameters returned to the baseline during withdrawal (Auta et al., 2017). It is worth adding that a similar study was conducted with the use of human cerebellum samples from AUD patients (Gatta et al., 2017). The decreased level of SAH, as well as higher ratio of SAM/SAH, in the AUD group was found as compared to the control cohort. However the global levels of 5mC and 5hmC were not changed in AUD patients (Gatta et al., 2017).

Conclusions: Many studies demonstrated differential DNA methylation in the brain reward system (mPFC, NAc, VTA, amygdala and cerebellum) of the animals exposed to alcohol, as compared to the alcohol-naives (Supplementary Table 1). In particular, the authors focused on the genes related to synaptic plasticity, as unbiased EWAS analyses for the murine brain were not available till 2021. Most of the studies relate to alcohol consumption during free access without demonstration of the causative role of the methylation in the behavior. Up to date there are only a few observations that link DNA methylation with AUD-related behaviors beyond free access alcohol consumption, including the correlation of DNA methylation with compulsive alcohol drinking (Barbier et al., 2015), anxiolysis (Krishnan et al., 2022) and alcohol seeking induced by alcohol-predicting cues (Pagano et al., 2022).

### 4.2. Human studies

While exploratory methylome-wide analyses in the human brain found no global differences between alcohol dependent and control subjects (Manzardo et al., 2012; Clark et al., 2022), the focused approaches were more successful. The authors tested the hypothesis that methylation of *prodynorphin (Pdyn)* CpG-SNPs in the PFC is associated with alcohol misuse (Taqi et al., 2011). Three *Pdyn* CpG-SNPs associated with AUD were found to be differentially methylated in the human brain. In the PFC of the patients, methylation levels of the C, non-risk variant of 3′-untranslated region (3′-UTR) SNP (rs2235749; C > T), were increased and positively correlated with dynorphins. The findings suggest a causal link between alcoholism-associated *Pdyn* 3′-UTR CpG-SNP methylation, activation of *Pdyn* transcription and vulnerability of individuals with the C non-risk allele(s) to develop AUD. One of the most comprehensive analyses exploring the role of DNA methylation in PFC and NAc in alcohol addiction-related behavior was conducted by Meng et al. (2021). The authors presented *in vivo* and *in vitro* studies with the use of human brain tissue and blood samples as well as cell cultures and transgenic mice. EWAS analysis of both brain and blood samples indicated *DLG associated protein 2 (Dlgap2)* gene as the most differentially methylated region associated with AUD. *Dlgap2* gene encodes a membrane- associated protein, whose functions include synapse organization and signaling in neurons. Next, the analysis on the sorted neuronal cells confirmed the observations and proved that the EWAS findings were not a derivative of cellular heterogeneity. Furthermore, authors observed an increased expression of *Dlgap2* gene with hypomethylation of its DMR *in vitro*. Additionally, *Dlgap2* KO mice showed lower alcohol consumption (Meng et al., 2021).

In the recent study using EWAS, five brain regions [anterior cingulate cortex (ACC), Brodmann Area 9 (BA9), caudate nucleus (CN), ventral striatum (VS) and putamen] were used to identify differentially methylated CpGs in AUD patients, as compared to the controls (Zillich et al., 2021). Differentially methylated cytosines were found only in CN and VS. There were no differentially methylated CpG sites in ACC, BA9 and putamen. The two hypomethylated sites in the CN were annotated to iron responsive element binding protein 2 (Ireb2) and 3-hydroxy-3-methylglutaryl-CoA reductase (Hmgcr). Eighteen sites were found in the VS, both hypo- and hypermethylated. The three most differentiated were annotated with solute carrier family 30 member 8 (*Slc30A8)*, glycosaminoglycan xylosylkinase (*Fam20B)* and prostate cancer associated transcript 29 (*Pcat29)*. Further analysis of gene ontologies allowed to indicate “homophilic cell adhesion *via* plasma-membrane adhesion molecules” and “cell-cell adhesion *via* plasma-membrane adhesion molecules” as the most overrepresented in the CN, and “Lsm1-7-Pat1 complex” in VS. (Zillich et al., 2021). In the following study the same group focused on the CN, VS, and putamen (PUT) (Zillich et al., 2022). The authors analyzed not only DNA methylation but also compared the methylation data with mRNA expression. Weighted correlation network analysis (WGCNA) was performed for DNA-methylation and gene expression data and gene overlap was tested. The WGCNA modules most strongly associated with AUD showed strong enrichment for immune response and inflammation pathways.

Another EWAS analysis of precuneus (the medial aspect of the posterior parietal lobe) and putamen from the AUD patients has indicated hypermethylated gene networks that included: “cell-to-cell signaling and interaction,” “nervous system development and function” as well as “neurodevelopmental and hereditary disorders,” while hypomethylated gene networks covered: “small molecule biochemistry” as well as “neurodevelopmental and neurological disorders” (Hagerty et al., 2016). In the EWAS performed with the use of human PFC samples of European Australians diagnosed with AUD and healthy controls (Wang et al., 2016), several differentially methylated genes were identified as associated with AUD, and around 70% of them were hypermethylated. However, the significant results (that withstood correction for multiple testing) were obtained only for the male group. The top hits genes with differentially methylated CpGs in promoters were: *complexin 2* (*Cplx2), U42A small nucleolar RNA (Snord42A), zinc finger homeobox 3 (Zfhx3)*, and *paternally expressed 10 (Peg10)* (Wang et al., 2016).

Results of GABAergic neurotransmission studies, conducted by Gatta and collaborators (Gatta et al., 2021a,b), support the hypothesis that changes in DNA methylation patterns observed in cerebellum may be involved in pathophysiology of AUD. Furthermore, altered DNA methylation of *NR3C1* gene associated with its reduced mRNA and protein levels was also indicated as playing an essential role in pathophysiology of AUD (Gatta et al., 2021a).

Conclusions: Global analyses of the human brain tissue with EWAS are still infrequent. Therefore, the conclusions about the DNA methylation patterns that are AUD-specific, and conserved across different human populations, cannot be drawn yet. Furthermore, one has to keep in mind that the EWAS do not allow for recognition between 5mC and 5hmC (Wang et al., 2020). Strikingly, most of the genes and pathways identified to be differentially methylated in the AUD brain (e.g., “homophilic cell adhesion *via* plasma-membrane adhesion molecules” and “cell-cell adhesion *via* plasma-membrane adhesion molecules” as the most overrepresented in the CN, and “Lsm1-7-Pat1 complex”) have been neglected in the animal studies that mostly focus on the genes related to synaptic plasticity (Supplementary Table 1). Hence, these pathways require future mechanistic studies in animal models. Also the role of the genes found to be differentially methylated in the context of alcohol misuse is mostly unknown, with rare exceptions such as *Dlgap2* (Meng et al., 2021). Finally, one has to note that the observed alterations of DNA methylation patterns in human AUD patients may be the consequence, rather than the cause of the disease.

## 5. Factors affecting alcohol misuse and DNA methylation status in the brain

Human data indicate that social and environmental factors, such as stress, trauma or alcohol misuse by parents, contribute to AUD propensity (Nylander and Roman, 2013; Pucci et al., 2019; Zheng et al., 2021; van den Oord et al., 2022). In the following section we review the literature testing the link between these factors, DNA methylation and AUD-related behaviors.

### 5.1. Prenatal and early life stress

Prenatal stress induces behavioral deficits in adult offspring of stressed dams such as increased anxiety, hyperlocomotion, stereotypic behaviors, social and memory deficits (Weinstock, 2017). There is also accumulating evidence that anxiety is one of the main factors promoting alcohol drinking (Murgatroyd et al., 2009; Davidson and McEwen, 2012; Krishnan et al., 2014). The training with repeated episodes of restraint stress of dames was used to explore the association between prenatal stress and epigenetic modification in the mPFC (Dong et al., 2016, 2018). Prenatally stressed mice showed more anxiety-like behaviors and higher ethanol intake in adulthood, as compared to the non-stressed control mice. Authors focused on the epigenetic modifications in the genes linked with synaptic plasticity and dendritic spine formation. There was also a significant increase in the expression of *DNMT1* and *DNMT3a* in the mPFC (Dong et al., 2016). Since stress is associated with impaired synaptic functions in mPFC (Davidson and McEwen, 2012), to find a mechanism of behavioral phenotype, genes related to synaptic formation and function were studied [*Arc*, *spinophilin* (*Spn*), *postsynaptic density 95* (*Psd95*), and *tropomyosin receptor kinase B (TrkB)*]. Significant enrichment of 5mC was found in the promoter region of *Arc* and *Spn* in the prenatally stressed mice. mRNA and protein levels of all mentioned genes were significantly decreased in these mice compared to the controls. In addition, prenatally stressed mice, as compared to non-stressed controls, had lower dendritic spine density (by about 30%) in mPFC. Thus, prenatal stress induces epigenetic mechanisms that alter alcohol consumption in adult life. However, the mechanistic link between these two phenomena still has to be established.

Adverse experiences in childhood significantly increase propensity for alcohol misuse later in life, both in humans and laboratory animals (Nylander and Roman, 2013). Vrettou et al. (2017) investigated the associations between the early-life stress (maternal separation) and the adult voluntary alcohol drinking in rats, as well as the expression of glutamatergic genes [vesicular glutamate transporters (*Vglut1-3*), *DNMT1* and *MeCP2*] in the brain reward circuit. Early-life stress was associated with down-regulated expression of Vglut2 in the VTA and mPFC. Moreover, the rats exposed to early-life stress were more sensitive to ethanol-induced changes of Vglut expression in the VTA, NAc, and dorsal striatum, and DNMT1 and Mecp2 expression in the striatal regions. These findings suggest long-term neuroadaptations as a consequence of early-life stress, and show an association between Vglut, Dnmt1 and Mecp2 expression and voluntary drinking in non-preferring, non-dependent, rodents with the history of early-life stress (Vrettou et al., 2017). In the following study, the authors asked whether the observed changes in Vglut1-3 expression were associated with the changes of DNA methylation. In the VTA, early-life stress was associated with Vglut1-2 CpG-specific hypomethylation in ethanol-drinking rats, whereas bidirectional Vglut2 methylation differences at single CpGs were associated with early-life stress alone. In the NAc, exposure to both early-life stress and ethanol was associated with lower promoter and higher intronic Vglut3 methylation; and in the dorsal striatum with higher (26% of CpGs) or lower (43% of CpGs) methylation of Vglut1 CpGs. In the mPFC, lower Vglut2 methylation was observed upon exposure to early-life stress or ethanol. These findings suggest that Vglut1-3 CpG-specific methylation in mPFC, NAc and dorsal striatum is a signature of early-life stress and ethanol drinking (Vrettou et al., 2021).

### 5.2. Inheritance of DNA methylation

DNA methylation is considered as a carrier of non-genetic information affected by environmental factors like stress, trauma (Youssef, 2022) or nutritional deprivations (Liberman et al., 2019). It is suggested that these adverse experiences may have an impact on animal behavior across generations by transmission of DNA methylation patterns from parents to offspring (Bohacek and Mansuy, 2013). Accordingly, the experience of parents may have a potential effect on DNA methylation and AUD propensity of their offspring.

The influences of alcohol exposure of male rats (sires) on behavioral and epigenetic changes in the offspring was studied by just a few groups. Although, in human studies alcohol use by parents is positively correlated with propensity for alcohol misuse by their children (Zheng et al., 2021; Ji and An, 2022), in rats chronic alcohol consumption by parents protected offspring against alcohol misuse (Finegersh and Homanics, 2014; Nieto et al., 2022). Furthermore, in the offspring of alcohol treated sires, the methylation pattern of *Bdnf* promoter in NAc and VTA was significantly changed as compared to the controls. In NAc, the alcohol-sired offspring males showed differential methylation levels in CpG 11 and 62 (hypomethylation) as well as CpG 43 (hypermethylation). In females, CpG 24 was hypomethylated while CpG 141 was hypermethylated. Ethanol exposure also decreased DNA methylation at the Bdnf promoter of sire’s germ cells and hypomethylation was maintained in the VTA of both male and female ethanol-sired offspring (Finegersh and Homanics, 2014). Thus, paternal alcohol consumption causes long-lasting changes in *Bdnf* DNA methylation levels, which are then transmitted to offspring of both sexes. The large number of hyper- and hypomethylated CpGs was also found in the hypothalamus of the rats with one or both parents exposed to alcohol (Asimes et al., 2017). Thus, there are reasons to argue that paternal alcohol exposure has intergenerational consequences but further research is necessary to investigate the mechanism and validate whether discovered DNA methylation changes indeed contribute to alcohol use by the offspring.

## 6. Summary, conclusions, and perspectives

We reviewed the literature with the aim to look for the link between the changes of DNA methylation status in the brain and AUD. Accumulating studies observe differential DNA methylation and expression of DNMTs in the brain reward system of the individuals (AUD patients, primates and rodents) exposed to alcohol (Supplementary Table 1). These molecular changes are specific for cell types and brain regions. Moreover, inhibition of DNMTs (systemic, intracerebroventricular or local in PFC) consistently reduced alcohol consumption. Hence, the data support the significant role of alcohol consumption in the regulation of DNA methylation and *vice versa*, and point at the possible use of DNMT inhibitors as alternative strategies for AUD therapy (Pucci et al., 2019; Rodriguez, 2021). Such developments are crucial as the therapies available so far are effective only for patients and burdened with side effects (Bourguet et al., 2018; Witkiewitz et al., 2019). Unfortunately, DNMT inhibitors FDA-approved for the treatment of myelodysplastic syndrome and acute myeloid leukemia (azacytidine and decitabine) also have high toxicity and serious side effects of these drugs include increased bruising, bleeding, and infection (Savarese and Lasek, 2018). Hence, further studies are needed on newer generation compounds, such as zebularine and RG-108 (Barbier et al., 2015).

The role of DNA methylation in the regulation of AUD-related behaviors beyond quantitative aspects of alcohol consumption, such as motivation to drink, alcohol craving, compulsive alcohol seeking and drinking or propensity for relapse, remain largely unknown, with rare exception where gene methylation was linked with compulsive alcohol use (Barbier et al., 2015), anxiolysis (Krishnan et al., 2022) or alcohol motivation and seeking (Pagano et al., 2022). Furthermore, the effect of alcohol use on the expression of the enzymes regulating DNA demethylation (TET and Alkb) in the brain still remain mostly unknown.

As social and environmental factors contribute to AUD propensity (Nylander and Roman, 2013; Zheng et al., 2021), we aimed to look for the link between those factors, DNA methylation and AUD etiology. Although a very broad range of stimuli affect DNA methylation (Sharma, 2015; Szyf, 2015; Bierer et al., 2020; Daskalakis et al., 2021), we conclude that the existing literature very poorly supports the role of DNA methylation in mediating the link between environmental and psychological factors and AUD propensity. Just a few studies correlate social factors, DNA methylation and alcohol use. Hence, future research should focus on understanding the role of those factors in regulation of brain region- and cell type-specific DNA methylation related to AUD-associated behaviors. Moreover, the important challenge is the functional validation of discovered effects- both at the levels of gene expression and regulation of AUD-related behaviors.

DNA methylation analyses of the brain tissue from human AUD patients are still very scarce, and rarely replicated in two cohorts. Moreover, the role of the differentially methylated genes identified in human patients, and their mechanistic contribution to the addiction process, is mostly unknown, with rare exceptions such as *Dlgap2* (Meng et al., 2021). Strikingly, most of the gene pathways found to be differentially methylated in the AUD brain, such as “homophilic cell adhesion *via* plasma-membrane adhesion molecules” and “cell-cell adhesion *via* plasma-membrane adhesion molecules” and “Lsm1-7-Pat1 complex” (Zillich et al., 2021), have been neglected in animal studies. However, to determine the function of these pathways in AUD etiology and regulation of AUD-related behaviors validation in animal models is required. On the other hand, DNA methylation studies in animal models focused mostly on the genes related to synaptic plasticity, such as *Syt2* (Barbier et al., 2015), *Bdnf*, *Npy* (Sakharkar et al., 2019), or *Gdnf* (Gatta et al., 2017), which were rarely replicated in patients. Thus, greater crosstalk between human studies and animal model-based analyses is required in the future.

In the context of AUD, DNA methylation analyses were conducted so far using either the brain tissue of AUD patients or the animals with the alcohol exposure history, but without AUD diagnosis. Unfortunately, these approaches have important limitations. By analyzing human brain tissue, one cannot distinguish between the DNA methylation patterns which contribute to the development of AUD and those that are altered by alcohol consumption or observed only at the advanced stages of the disease. On the other hand, when simple animal models are used (without identification of AUD-prone individuals) it remains unknown whether observed changes of DNA methylation are related to ethanol exposure or AUD progression. Thus, one of the important points in the future studies aiming at understanding the role of DNA methylation in AUD, is the need to use more advanced animal models which characterize AUD phenotype based on DSM-5 criteria of the disease and therefore indicate AUD-prone individuals. Such an approach will distinguish the DNA methylation events that are induced by ethanol exposure from those that control AUD proness and progression.

Finally, DNA methylation studies also face important technical limitations. Commonly used platforms only capture a small percentage of the methylome (e.g., Illumina 450k, < 2%) and typically focus on CpG-rich “islands” near promoter regions—as such, many regions of potential relevance to addiction remain largely inaccessible.

**FIGURE 3:**
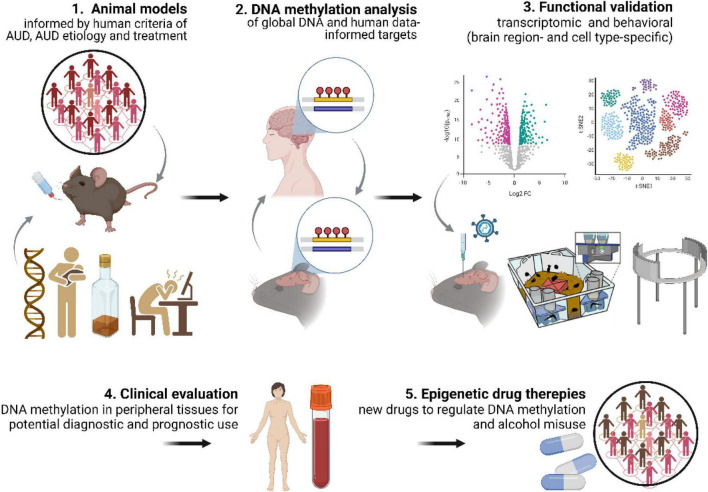
DNA methylation discovery pipeline. Key aspects needed to increase contribution of DNA methylation studies to understanding AUD mechanisms include: (1) use of advanced, multidimensional animal models of AUD; (2) DNA methylation analyses in animal models informed by human data (and *vice versa*); (3) functional validation of DNA methylation both at the transcriptomic and behavioral levels. This approach will allow for (4) identification of DNA methylation markers with diagnostic and prognostic potential and (5) development of epigenetic drug therapies.

Overall, we conclude that our comprehension of the role of DNA methylation and repair in AUD etiology and progression would greatly benefit if future studies took into consideration: (1) well validated animal models of AUD; (2) understanding how differentially methylated genes identified in the human EWAS contribute to addiction process; (3) testing whether DNA methylation contributes not only to alcohol consumption but also other AUD-related behaviors such as motivation to drink, alcohol craving or propensity for relapse; (4) testing the role of DNA methylation in mediating the role of social and environmental factors in AUD propensity; (5) experimental validation of DNA methylation changes at the levels of gene expression and regulation of behavior (Figure 3). The continued systematic investigation of DNA methylation using emerging techniques is likely to lead to further insights into DNA methylation biology and will continue to unravel the mechanisms related to AUD etiology. Such findings will be crucial for development of DNA methylation markers for AUD diagnosis and prognosis as well as alternative epigenetic drug therapies (Rodriguez, 2021).

## Author contributions

JJ drafted the manuscript. JJ, MM, and KR worked together toward its final version. All authors contributed to the article and approved the submitted version.

## References

[B1] American Psychiatric Association (2013). *Diagnostic and statistical manual of mental disorders: DSM-5*, 5th Edn. Washington, D.C: American Psychiatric Association.

[B2] Amir R. E. Van den Veyver I. B. Wan M. Tran C. Q. Francke U. Zoghbi H. Y. (1999). Rett syndrome is caused by mutations in X-linked MECP2, encoding methyl-CpG-binding protein 2. *Nat. Genet.* 23 185–188. 10.1038/13810 10508514

[B3] Asimes A. Torcaso A. Pinceti E. Kim C. K. Zeleznik-Le N. J. Pak T. R. (2017). Adolescent binge-pattern alcohol exposure alters genome-wide DNA methylation patterns in the hypothalamus of alcohol-naïve male offspring. *Alcohol* 60 179–189. 10.1016/j.alcohol.2016.10.010 27817987 PMC5403620

[B4] Aspra Q. Cabrera-Mendoza B. Morales-Marín M. E. Márquez C. Chicalote C. Ballesteros A. (2022). Epigenome-wide analysis reveals DNA methylation alteration in ZFP57 and its target RASGFR2 in a mexican population cohort with autism. *Child. Basel Switz.* 9:462. 10.3390/children9040462 35455506 PMC9025761

[B5] Ausió J. (2016). MeCP2 and the enigmatic organization of brain chromatin. Implications for depression and cocaine addiction. *Clin. Epigenetics* 8:58. 10.1186/s13148-016-0214-5 27213019 PMC4875624

[B6] Auta J. Zhang H. Pandey S. C. Guidotti A. (2017). Chronic alcohol exposure differentially alters one-carbon metabolism in rat liver and brain. *Alcohol. Clin. Exp. Res.* 41 1105–1111. 10.1111/acer.13382 28369960 PMC5494979

[B7] Barbier E. Tapocik J. D. Juergens N. Pitcairn C. Borich A. Schank J. R. (2015). DNA methylation in the medial prefrontal cortex regulates alcohol-induced behavior and plasticity. *J. Neurosci.* 35 6153–6164. 10.1523/JNEUROSCI.4571-14.2015 25878287 PMC4397609

[B8] Bendre M. Granholm L. Drennan R. Meyer A. Yan L. Nilsson K. W. (2019). Early life stress and voluntary alcohol consumption in relation to maoa methylation in male rats. *Alcohol* 79 7–16. 10.1016/j.alcohol.2018.11.001 30414913

[B9] Berkel T. D. M. Pandey S. C. (2017). Emerging role of epigenetic mechanisms in alcohol addiction. *Alcohol. Clin. Exp. Res.* 41 666–680. 10.1111/acer.13338 28111764 PMC5378655

[B10] Beroun A. Nalberczak-Skóra M. Harda Z. Piechota M. Ziółkowska M. Cały A. (2018). Generation of silent synapses in dentate gyrus correlates with development of alcohol addiction. *Neuropsychopharmacology* 43 1989–1999. 10.1038/s41386-018-0119-4 29967367 PMC6098144

[B11] Bian K. Lenz S. A. P. Tang Q. Chen F. Qi R. Jost M. (2019). DNA repair enzymes ALKBH2, ALKBH3, and AlkB oxidize 5-methylcytosine to 5-hydroxymethylcytosine, 5-formylcytosine and 5-carboxylcytosine in vitro. *Nucleic Acids Res.* 47 5522–5529. 10.1093/nar/gkz395 31114894 PMC6582317

[B12] Bierer L. M. Bader H. N. Daskalakis N. P. Lehrner A. Provençal N. Wiechmann T. (2020). Intergenerational effects of maternal holocaust exposure on FKBP5 methylation. *Am. J. Psychiatry* 177 744–753. 10.1176/appi.ajp.2019.19060618 32312110

[B13] Bierut L. J. Dinwiddie S. H. Begleiter H. Crowe R. R. Hesselbrock V. Nurnberger J. I. (1998). Familial transmission of substance dependence: Alcohol, marijuana, cocaine, and habitual smoking: A report from the collaborative study on the genetics of alcoholism. *Arch. Gen. Psychiatry* 55 982–988. 10.1001/archpsyc.55.11.982 9819066

[B14] Bin Akhtar G. Buist M. Rastegar M. (2022). MeCP2 and transcriptional control of eukaryotic gene expression. *Eur. J. Cell Biol.* 101:151237. 10.1016/j.ejcb.2022.151237 35588541

[B15] Bohacek J. Mansuy I. M. (2013). Epigenetic inheritance of disease and disease risk. *Neuropsychopharmacology* 38 220–236. 10.1038/npp.2012.110 22781843 PMC3521963

[B16] Bourguet E. Ozdarska K. Moroy G. Jeanblanc J. Naassila M. (2018). Class I HDAC inhibitors: Potential new epigenetic therapeutics for alcohol use disorder (AUD). *J. Med. Chem.* 61 1745–1766. 10.1021/acs.jmedchem.7b00115 28771357

[B17] Brenet F. Moh M. Funk P. Feierstein E. Viale A. J. Socci N. D. (2011). DNA methylation of the first exon is tightly linked to transcriptional silencing. *PLoS One* 6:e14524. 10.1371/journal.pone.0014524 21267076 PMC3022582

[B18] Carvalho A. F. Heilig M. Perez A. Probst C. Rehm J. (2019). Alcohol use disorders. *Lancet* 394 781–792. 10.1016/S0140-6736(19)31775-1 31478502

[B19] Cervera-Juanes R. Wilhelm L. J. Park B. Grant K. A. Ferguson B. (2017). Alcohol-dose-dependent DNA methylation and expression in the nucleus accumbens identifies coordinated regulation of synaptic genes. *Transl. Psychiatry* 7:e994. 10.1038/tp.2016.266 28072409 PMC5545731

[B20] Chen Z. Riggs A. D. (2011). DNA methylation and demethylation in mammals. *J. Biol. Chem.* 286 18347–18353. 10.1074/jbc.R110.205286 21454628 PMC3099650

[B21] Cholewa-Waclaw J. Bird A. von Schimmelmann M. Schaefer A. Yu H. Song H. (2016). The role of epigenetic mechanisms in the regulation of gene expression in the nervous system. *J. Neurosci.* 36 11427–11434. 10.1523/JNEUROSCI.2492-16.2016 27911745 PMC5125210

[B22] Clark S. L. Chan R. F. Zhao M. Xie L. Y. Copeland W. E. Penninx B. W. J. H. (2022). Dual methylation and hydroxymethylation study of alcohol use disorder. *Addict. Biol.* 27:e13114. 10.1111/adb.13114 34791764 PMC8891051

[B23] Cui H. Z. Sun M. Z. Wang R. Z. Li C. Y. Huang Y. X. Huang Q. J. (2020). DNA methylation in the medial prefrontal cortex regulates alcohol-related behavior in rats. *Yi Chuan Hered.* 42 112–125. 10.16288/j.yczz.19-261 31956101

[B24] Daskalakis N. P. Xu C. Bader H. N. Chatzinakos C. Weber P. Makotkine I. (2021). Intergenerational trauma is associated with expression alterations in glucocorticoid- and immune-related genes. *Neuropsychopharmacology* 46 763–773. 10.1038/s41386-020-00900-8 33173192 PMC8027026

[B25] Davidson R. J. McEwen B. S. (2012). Social influences on neuroplasticity: Stress and interventions to promote well-being. *Nat. Neurosci.* 15 689–695. 10.1038/nn.3093 22534579 PMC3491815

[B26] De Sa Nogueira D. Merienne K. Befort K. (2019). Neuroepigenetics and addictive behaviors: Where do we stand? *Neurosci. Biobehav. Rev.* 106 58–72. 10.1016/j.neubiorev.2018.08.018 30205119

[B27] Dong E. Guidotti A. Zhang H. Pandey S. C. (2018). Prenatal stress leads to chromatin and synaptic remodeling and excessive alcohol intake comorbid with anxiety-like behaviors in adult offspring. *Neuropharmacology* 140 76–85. 10.1016/j.neuropharm.2018.07.010 30016666 PMC6499375

[B28] Dong E. Tueting P. Matrisciano F. Grayson D. R. Guidotti A. (2016). Behavioral and molecular neuroepigenetic alterations in prenatally stressed mice: Relevance for the study of chromatin remodeling properties of antipsychotic drugs. *Transl. Psychiatry* 6:e711. 10.1038/tp.2015.191 26756904 PMC5068871

[B29] Egervari G. Siciliano C. A. Whiteley E. L. Ron D. (2021). Alcohol and the brain: From genes to circuits. *Trends Neurosci.* 44 1004–1015. 10.1016/j.tins.2021.09.006 34702580 PMC8616825

[B30] Finegersh A. Homanics G. E. (2014). Paternal alcohol exposure reduces alcohol drinking and increases behavioral sensitivity to alcohol selectively in male offspring. *PLoS One* 9:e99078. 10.1371/journal.pone.0099078 24896617 PMC4045990

[B31] Gatta E. Auta J. Gavin D. P. Bhaumik D. K. Grayson D. R. Pandey S. C. (2017). Emerging role of one-carbon metabolism and DNA methylation enrichment on δ-containing GABAA receptor expression in the cerebellum of subjects with alcohol use disorders (AUD). *Int. J. Neuropsychopharmacol.* 20 1013–1026. 10.1093/ijnp/pyx075 29020412 PMC5716183

[B32] Gatta E. Grayson D. R. Auta J. Saudagar V. Dong E. Chen Y. (2021a). Genome-wide methylation in alcohol use disorder subjects: Implications for an epigenetic regulation of the cortico-limbic glucocorticoid receptors (NR3C1). *Mol. Psychiatry* 26 1029–1041. 10.1038/s41380-019-0449-6 31239533 PMC6930366

[B33] Gatta E. Guidotti A. Saudagar V. Grayson D. R. Aspesi D. Pandey S. C. (2021b). Epigenetic regulation of GABAergic neurotransmission and neurosteroid biosynthesis in alcohol use disorder. *Int. J. Neuropsychopharmacol.* 24 130–141. 10.1093/ijnp/pyaa073 32968808 PMC7883893

[B34] Gavin D. P. Kusumo H. Zhang H. Guidotti A. Pandey S. C. (2016). Role of growth arrest and DNA damage-inducible, beta in alcohol-drinking behaviors. *Alcohol. Clin. Exp. Res.* 40 263–272. 10.1111/acer.12965 26842245 PMC4743544

[B35] Gelernter J. Polimanti R. (2021). Genetics of substance use disorders in the era of big data. *Nat. Rev. Genet.* 22 712–729. 10.1038/s41576-021-00377-1 34211176 PMC9210391

[B36] Gipson C. D. Kupchik Y. M. Shen H. Reissner K. J. Thomas C. A. Kalivas P. W. (2013). Relapse induced by cues predicting cocaine depends on rapid, transient synaptic potentiation. *Neuron* 77 867–872. 10.1016/j.neuron.2013.01.005 23473317 PMC3619421

[B37] Glantz M. D. Bharat C. Degenhardt L. Sampson N. A. Scott K. M. Lim C. C. W. (2020). The epidemiology of alcohol use disorders cross-nationally: Findings from the world mental health surveys. *Addict. Behav.* 102:106128. 10.1016/j.addbeh.2019.106128 31865172 PMC7416527

[B38] Goltseker K. Hopf F. W. Barak S. (2019). Advances in behavioral animal models of alcohol use disorder. *Alcohol* 74 73–82. 10.1016/j.alcohol.2018.05.014 30424979

[B39] Gowen A. M. Odegaard K. E. Hernandez J. Chand S. Koul S. Pendyala G. (2021). Role of microRNAs in the pathophysiology of addiction. *Wiley Interdiscip. Rev. RNA* 12:e1637. 10.1002/wrna.1637 33336550 PMC8026578

[B40] Guidotti A. Dong E. Gavin D. P. Veldic M. Zhao W. Bhaumik D. K. (2013). DNA methylation/demethylation network expression in psychotic patients with a history of alcohol abuse. *Alcohol. Clin. Exp. Res.* 37 417–424. 10.1111/j.1530-0277.2012.01947.x 22958170

[B41] Hagerty S. L. Bidwell L. C. Harlaar N. Hutchison K. E. (2016). An exploratory association study of alcohol use disorder and DNA methylation. *Alcohol. Clin. Exp. Res.* 40 1633–1640. 10.1111/acer.13138 27388583 PMC5108727

[B42] Hardwick J. S. Lane A. N. Brown T. (2018). Epigenetic modifications of cytosine: Biophysical properties, regulation, and function in mammalian DNA. *Bioessays* 40:1700199. 10.1002/bies.201700199 29369386

[B43] Hwang J.-Y. Aromolaran K. A. Zukin R. S. (2017). The emerging field of epigenetics in neurodegeneration and neuroprotection. *Nat. Rev. Neurosci.* 18 347–361. 10.1038/nrn.2017.46 28515491 PMC6380351

[B44] Ji M. An R. (2022). Parental effects on obesity, smoking, and drinking in children and adolescents: A twin study. *J. Adolesc. Health* 71 196–203. 10.1016/j.jadohealth.2022.02.016 35550332

[B45] Kaut O. Schmitt I. Stahl F. Fröhlich H. Hoffmann P. Gonzalez F. J. (2022). Epigenome-wide analysis of DNA methylation in Parkinson’s disease cortex. *Life Basel Switz.* 12:502. 10.3390/life12040502 35454993 PMC9025601

[B46] Keistler C. R. Hammarlund E. Barker J. M. Bond C. W. DiLeone R. J. Pittenger C. (2017). Regulation of alcohol extinction and cue-induced reinstatement by specific projections among medial prefrontal cortex, nucleus accumbens, and basolateral amygdala. *J. Neurosci.* 37 4462–4471. 10.1523/JNEUROSCI.3383-16.2017 28336571 PMC5413184

[B47] Kirsch D. E. Lippard E. T. C. (2022). Early life stress and substance use disorders: The critical role of adolescent substance use. *Pharmacol. Biochem. Behav.* 215:173360. 10.1016/j.pbb.2022.173360 35219756 PMC8983562

[B48] Kohli R. M. Zhang Y. (2013). TET enzymes, TDG and the dynamics of DNA demethylation. *Nature* 502 472–479. 10.1038/nature12750 24153300 PMC4046508

[B49] Kozlenkov A. Jaffe A. E. Timashpolsky A. Apontes P. Rudchenko S. Barbu M. (2017). DNA methylation profiling of human prefrontal cortex neurons in heroin users shows significant difference between genomic contexts of hyper–and hypomethylation and a younger epigenetic age. *Genes* 8:E152. 10.3390/genes8060152 28556790 PMC5485516

[B50] Kranzler H. R. Zhou H. Kember R. L. Vickers Smith R. Justice A. C. Damrauer S. (2019). Genome-wide association study of alcohol consumption and use disorder in 274,424 individuals from multiple populations. *Nat. Commun.* 10:1499. 10.1038/s41467-019-09480-8 30940813 PMC6445072

[B51] Krishnan H. R. Sakharkar A. J. Teppen T. L. Berkel T. D. M. Pandey S. C. (2014). The epigenetic landscape of alcoholism. *Int. Rev. Neurobiol.* 115 75–116. 10.1016/B978-0-12-801311-3.00003-2 25131543 PMC4337828

[B52] Krishnan H. R. Zhang H. Chen Y. Bohnsack J. P. Shieh A. W. Kusumo H. (2022). Unraveling the epigenomic and transcriptomic interplay during alcohol-induced anxiolysis. *Mol. Psychiatry* 27 4624–4632. 10.1038/s41380-022-01732-2 36089615 PMC9734037

[B53] Liberman N. Wang S. Y. Greer E. L. (2019). Transgenerational epigenetic inheritance: From phenomena to molecular mechanisms. *Curr. Opin. Neurobiol.* 59 189–206. 10.1016/j.conb.2019.09.012 31634674 PMC6889819

[B54] Liyanage V. R. B. Curtis K. Zachariah R. M. Chudley A. E. Rastegar M. (2017). Overview of the genetic basis and epigenetic mechanisms that contribute to FASD pathobiology. *Curr. Top. Med. Chem.* 17 808–828. 10.2174/1568026616666160414124816 27086780

[B55] Longley M. J. Lee J. Jung J. Lohoff F. W. (2021). Epigenetics of alcohol use disorder–a review of recent advances in DNA methylation profiling. *Addict. Biol.* 26:e13006. 10.1111/adb.13006 33538087 PMC8596445

[B56] Maier H. B. Neyazi M. Neyazi A. Hillemacher T. Pathak H. Rhein M. (2020). Alcohol consumption alters Gdnf promoter methylation and expression in rats. *J. Psychiatr. Res.* 121 1–9. 10.1016/j.jpsychires.2019.10.020 31710958

[B57] Manzardo A. M. Henkhaus R. S. Butler M. G. (2012). Global DNA promoter methylation in frontal cortex of alcoholics and controls. *Gene* 498 5–12. 10.1016/j.gene.2012.01.096 22353363 PMC3653411

[B58] Meng W. Sjöholm L. K. Kononenko O. Tay N. Zhang D. Sarkisyan D. (2021). Genotype-dependent epigenetic regulation of DLGAP2 in alcohol use and dependence. *Mol. Psychiatry* 26 4367–4382. 10.1038/s41380-019-0588-9 31745236

[B59] Midanik L. (1983). Familial alcoholism and problem drinking in a national drinking practices survey. *Addict. Behav.* 8 133–141. 10.1016/0306-4603(83)90007-2 6613712

[B60] Montalvo-Ortiz J. L. Gelernter J. Cheng Z. Girgenti M. J. Xu K. Zhang X. (2022). Epigenome-wide association study of posttraumatic stress disorder identifies novel loci in U.S. military veterans. *Transl. Psychiatry* 12:65. 10.1038/s41398-022-01822-3 35177594 PMC8854688

[B61] Murgatroyd C. Patchev A. V. Wu Y. Micale V. Bockmühl Y. Fischer D. (2009). Dynamic DNA methylation programs persistent adverse effects of early-life stress. *Nat. Neurosci.* 12 1559–1566. 10.1038/nn.2436 19898468

[B62] Nabel C. S. Manning S. A. Kohli R. M. (2012). The curious chemical biology of cytosine: Deamination, methylation, and oxidation as modulators of genomic potential. *ACS Chem. Biol.* 7 20–30. 10.1021/cb2002895 22004246 PMC3262930

[B63] Nalberczak-Skóra M. Pattij T. Beroun A. Kogias G. Mielenz D. de Vries T. (2020). Personality driven alcohol and drug abuse: New mechanisms revealed. *Neurosci. Biobehav. Rev.* 116 64–73. 10.1016/j.neubiorev.2020.06.023 32565173

[B64] Namba M. D. Tomek S. E. Olive M. F. Beckmann J. S. Gipson C. D. (2018). The winding road to relapse: Forging a new understanding of cue-induced reinstatement models and their associated neural mechanisms. *Front. Behav. Neurosci.* 12:17. 10.3389/fnbeh.2018.00017 29479311 PMC5811475

[B65] Nestler E. J. Lüscher C. (2019). The molecular basis of drug addiction: Linking epigenetic to synaptic and circuit mechanisms. *Neuron* 102 48–59. 10.1016/j.neuron.2019.01.016 30946825 PMC6587180

[B66] Nielsen D. A. Utrankar A. Reyes J. A. Simons D. D. Kosten T. R. (2012). Epigenetics of drug abuse: Predisposition or response. *Pharmacogenomics* 13 1149–1160. 10.2217/pgs.12.94 22909205 PMC3463407

[B67] Nieto S. J. Haile C. N. Quave C. B. Harding M. J. Nielsen D. A. Meisch R. A. (2022). Paternal alcohol exposure reduces acquisition of operant alcohol self-administration and affects Bdnf DNA methylation in male and female offspring. *Addict. Biol.* 27:e13078. 10.1111/adb.13078 34363290 PMC8720057

[B68] Niinep K. Anier K. Eteläinen T. Piepponen P. Kalda A. (2021). Repeated ethanol exposure alters DNA methylation Status and dynorphin/kappa-opioid receptor expression in nucleus accumbens of alcohol-preferring AA rats. *Front. Genet.* 12:750142. 10.3389/fgene.2021.750142 34899839 PMC8652212

[B69] Nylander I. Roman E. (2013). Is the rodent maternal separation model a valid and effective model for studies on the early-life impact on ethanol consumption? *Psychopharmacology (Berl)* 229 555–569. 10.1007/s00213-013-3217-3 23982922 PMC3782650

[B70] Pagano R. Salamian A. Zielinski J. Beroun A. Nalberczak-Skóra M. Skonieczna E. (2022). Arc controls alcohol cue relapse by a central amygdala mechanism. *Mol. Psychiatry* 1–13. 10.1038/s41380-022-01849-4 [Epub ahead of print]. 36357670

[B71] Palmisano M. Pandey S. C. (2017). Epigenetic mechanisms of alcoholism and stress-related disorders. *Alcohol* 60 7–18. 10.1016/j.alcohol.2017.01.001 28477725 PMC5464725

[B72] Perry G. S. Das M. Woon E. C. Y. (2021). Inhibition of AlkB Nucleic acid demethylases: Promising new epigenetic targets. *J. Med. Chem.* 64 16974–17003. 10.1021/acs.jmedchem.1c01694 34792334

[B73] Ponomarev I. (2013). Epigenetic control of gene expression in the alcoholic brain. *Alcohol Res. Curr. Rev.* 35 69–76. 10.35946/arcr.v35.1.08 PMC3860426 24313166

[B74] Pucci M. Micioni Di Bonaventura M. V. Wille-Bille A. Fernández M. S. Maccarrone M. Pautassi R. M. (2019). Environmental stressors and alcoholism development: Focus on molecular targets and their epigenetic regulation. *Neurosci. Biobehav. Rev.* 106 165–181. 10.1016/j.neubiorev.2018.07.004 30017749

[B75] Qiang M. Denny A. D. Ticku M. K. (2007). Chronic intermittent ethanol treatment selectively alters N-methyl-D-aspartate receptor subunit surface expression in cultured cortical neurons. *Mol. Pharmacol.* 72 95–102. 10.1124/mol.106.033043 17440117

[B76] Qiang M. Denny A. Chen J. Ticku M. K. Yan B. Henderson G. (2010). The site specific demethylation in the 5′-regulatory area of NMDA receptor 2B subunit gene associated with CIE-induced up-regulation of transcription. *PLoS One* 5:e8798. 10.1371/journal.pone.0008798 20098704 PMC2808353

[B77] Qiang M. Li J. G. Denny A. D. Yao J.-M. Lieu M. Zhang K. (2014). Epigenetic mechanisms are involved in the regulation of ethanol consumption in mice. *Int. J. Neuropsychopharmacol.* 18:yu072. 10.1093/ijnp/pyu072 25522411 PMC4368896

[B78] Qiao X. Yin F. Ji Y. Li Y. Yan P. Lai J. (2017). 5-Aza-2′-deoxycytidine in the medial prefrontal cortex regulates alcohol-related behavior and Ntf3-TrkC expression in rats. *PLoS One* 12:e0179469. 10.1371/journal.pone.0179469 28614398 PMC5470731

[B79] Rasmussen K. D. Helin K. (2016). Role of TET enzymes in DNA methylation, development, and cancer. *Genes Dev.* 30 733–750. 10.1101/gad.276568.115 27036965 PMC4826392

[B80] Rehm J. Shield K. D. (2019). Global burden of disease and the impact of mental and addictive disorders. *Curr. Psychiatry Rep.* 21:10. 10.1007/s11920-019-0997-0 30729322

[B81] Repunte-Canonigo V. Chen J. Lefebvre C. Kawamura T. Kreifeldt M. Basson O. (2014). MeCP2 regulates ethanol sensitivity and intake. *Addict. Biol.* 19 791–799. 10.1111/adb.12047 23448145 PMC3692583

[B82] Rodriguez F. D. (2021). Targeting epigenetic mechanisms to treat alcohol use disorders (AUD). *Curr. Pharm. Des.* 27 3252–3272. 10.2174/1381612827666210203142539 33535943 PMC8778698

[B83] Ross S. E. Bogdanovic O. (2019). TET enzymes, DNA demethylation and pluripotency. *Biochem. Soc. Trans.* 47 875–885. 10.1042/BST20180606 31209155

[B84] Rudenko A. Dawlaty M. M. Seo J. Cheng A. W. Meng J. Le T. (2013). Tet1 is critical for neuronal activity-regulated gene expression and memory extinction. *Neuron* 79 1109–1122. 10.1016/j.neuron.2013.08.003 24050401 PMC4543319

[B85] Rustad S. R. Papale L. A. Alisch R. S. (2019). DNA methylation and hydroxymethylation and behavior. *Curr. Top. Behav. Neurosci.* 42 51–82. 10.1007/7854_2019_104 31392630

[B86] Sakharkar A. J. Kyzar E. J. Gavin D. P. Zhang H. Chen Y. Krishnan H. R. (2019). Altered amygdala DNA methylation mechanisms after adolescent alcohol exposure contribute to adult anxiety and alcohol drinking. *Neuropharmacology* 157:107679. 10.1016/j.neuropharm.2019.107679 31229451 PMC6681823

[B87] Savarese A. M. Lasek A. W. (2018). “Transcriptional regulators as targets for alcohol pharmacotherapies,” in *The neuropharmacology of alcohol handbook of experimental pharmacology*, eds Grant K. A. Lovinger D. M. (Cham: Springer International Publishing), 505–533. 10.1007/164_2018_101 PMC6242703 29594350

[B88] Schmidt A. Zhang H. Cardoso M. C. (2020). MeCP2 and chromatin compartmentalization. *Cells* 9:E878. 10.3390/cells9040878 32260176 PMC7226738

[B89] Schumann G. Loth E. Banaschewski T. Barbot A. Barker G. Büchel C. (2010). The IMAGEN study: Reinforcement-related behaviour in normal brain function and psychopathology. *Mol. Psychiatry* 15 1128–1139. 10.1038/mp.2010.4 21102431

[B90] See R. E. (2002). Neural substrates of conditioned-cued relapse to drug-seeking behavior. *Pharmacol. Biochem. Behav.* 71 517–529. 10.1016/S0091-3057(01)00682-7 11830186

[B91] Sharma A. (2015). Transgenerational epigenetic inheritance requires a much deeper analysis. *Trends Mol. Med.* 21 269–270. 10.1016/j.molmed.2015.02.010 25795540

[B92] Shen L. Song C.-X. He C. Zhang Y. (2014). Mechanism and function of oxidative reversal of DNA and RNA methylation. *Annu. Rev. Biochem.* 83 585–614. 10.1146/annurev-biochem-060713-035513 24905787 PMC4786441

[B93] Song J. Chen Y. Zhao Q. Li H. Li W. Chen K. (2022). Leptin methylation and mRNA expression associated with psychopathology in schizophrenia inpatients. *Front. Psychiatry* 13:793910. 10.3389/fpsyt.2022.793910 35197874 PMC8858839

[B94] Stefaniuk M. Beroun A. Lebitko T. Markina O. Leski S. Meyza K. (2017). Matrix metalloproteinase-9 and synaptic plasticity in the central amygdala in control of alcohol-seeking behavior. *Biol. Psychiatry* 81 907–917. 10.1016/j.biopsych.2016.12.026 28190519

[B95] Szyf M. (2015). Nongenetic inheritance and transgenerational epigenetics. *Trends Mol. Med.* 21 134–144. 10.1016/j.molmed.2014.12.004 25601643

[B96] Taqi M. M. Bazov I. Watanabe H. Sheedy D. Harper C. Alkass K. (2011). Prodynorphin CpG-SNPs associated with alcohol dependence: Elevated methylation in the brain of human alcoholics. *Addict. Biol.* 16 499–509. 10.1111/j.1369-1600.2011.00323.x 21521424 PMC3391609

[B97] Tawa E. A. Hall S. D. Lohoff F. W. (2016). Overview of the genetics of alcohol use disorder. *Alcohol Alcohol.* 51 507–514. 10.1093/alcalc/agw046 27445363 PMC5004749

[B98] Tognini P. Napoli D. Pizzorusso T. (2015). Dynamic DNA methylation in the brain: A new epigenetic mark for experience-dependent plasticity. *Front. Cell. Neurosci.* 9:331. 10.3389/fncel.2015.00331 26379502 PMC4548453

[B99] Traube F. R. Carell T. (2017). The chemistries and consequences of DNA and RNA methylation and demethylation. *RNA Biol.* 14 1099–1107. 10.1080/15476286.2017.1318241 28440690 PMC5699545

[B100] Tulisiak C. T. Harris R. A. Ponomarev I. (2017). DNA modifications in models of alcohol use disorders. *Alcohol* 60 19–30. 10.1016/j.alcohol.2016.11.004 27865607 PMC5420490

[B101] van den Oord C. L. J. D. Copeland W. E. Zhao M. Xie L. Y. Aberg K. A. van den Oord E. J. C. G. (2022). DNA methylation signatures of childhood trauma predict psychiatric disorders and other adverse outcomes 17 years after exposure. *Mol. Psychiatry* 27 3367–3373. 10.1038/s41380-022-01597-5 35546634 PMC9649837

[B102] Verhulst B. Neale M. C. Kendler K. S. (2015). The heritability of alcohol use disorders: A meta-analysis of twin and adoption studies. *Psychol. Med.* 45 1061–1072. 10.1017/S0033291714002165 25171596 PMC4345133

[B103] Vrettou M. Granholm L. Todkar A. Nilsson K. W. Wallén-Mackenzie Å Nylander I. (2017). Ethanol affects limbic and striatal presynaptic glutamatergic and DNA methylation gene expression in outbred rats exposed to early-life stress. *Addict. Biol.* 22 369–380. 10.1111/adb.12331 26610727

[B104] Vrettou M. Yan L. Nilsson K. W. Wallén-Mackenzie Å Nylander I. Comasco E. (2021). DNA methylation of vesicular glutamate transporters in the mesocorticolimbic brain following early-life stress and adult ethanol exposure-an explorative study. *Sci. Rep.* 11:15322. 10.1038/s41598-021-94739-8 34321562 PMC8319394

[B105] Wan M. Lee S. S. Zhang X. Houwink-Manville I. Song H. R. Amir R. E. (1999). Rett syndrome and beyond: Recurrent spontaneous and familial MECP2 mutations at CpG hotspots. *Am. J. Hum. Genet.* 65 1520–1529. 10.1086/302690 10577905 PMC1288362

[B106] Wang F. Xu H. Zhao H. Gelernter J. Zhang H. (2016). DNA co-methylation modules in postmortem prefrontal cortex tissues of European Australians with alcohol use disorders. *Sci. Rep.* 6:19430. 10.1038/srep19430 26763658 PMC4725922

[B107] Wang Z. Du M. Yuan Q. Guo Y. Hutchinson J. N. Su L. (2020). Epigenomic analysis of 5-hydroxymethylcytosine (5hmC) reveals novel DNA methylation markers for lung cancers. *Neoplasia* 22 154–161. 10.1016/j.neo.2020.01.001 32062069 PMC7021546

[B108] Warnault V. Darcq E. Levine A. Barak S. Ron D. (2013). Chromatin remodeling–a novel strategy to control excessive alcohol drinking. *Transl. Psychiatry* 3:e231. 10.1038/tp.2013.4 23423140 PMC3591000

[B109] Weinstock M. (2017). Prenatal stressors in rodents: Effects on behavior. *Neurobiol. Stress* 6 3–13. 10.1016/j.ynstr.2016.08.004 28229104 PMC5314420

[B110] Witkiewitz K. Litten R. Z. Leggio L. (2019). Advances in the science and treatment of alcohol use disorder. *Sci. Adv.* 5:eaax4043. 10.1126/sciadv.aax4043 31579824 PMC6760932

[B111] Wojdacz T. K. Amarasinghe H. E. Kadalayil L. Beattie A. Forster J. Blakemore S. J. (2019). Clinical significance of DNA methylation in chronic lymphocytic leukemia patients: Results from 3 UK clinical trials. *Blood Adv.* 3 2474–2481. 10.1182/bloodadvances.2019000237 31434681 PMC6712529

[B112] Xu G.-L. Bochtler M. (2020). Reversal of nucleobase methylation by dioxygenases. *Nat. Chem. Biol.* 16 1160–1169. 10.1038/s41589-020-00675-5 33067602

[B113] Youssef N. A. (2022). Potential societal and cultural implications of transgenerational epigenetic methylation of trauma and PTSD: Pathology or resilience? *Yale J. Biol. Med.* 95 171–174. 35370497 PMC8961703

[B114] Zhang R.-R. Cui Q.-Y. Murai K. Lim Y. C. Smith Z. D. Jin S. (2013). Tet1 regulates adult hippocampal neurogenesis and cognition. *Cell Stem Cell* 13 237–245. 10.1016/j.stem.2013.05.006 23770080 PMC4474382

[B115] Zheng Y. Brendgen M. Meyer Z. Vitaro F. Dionne G. Boivin M. (2021). Maternal parenting behaviors amplify environmental influences on developmental trajectories of alcohol use during adolescence. *Behav. Genet.* 51 528–542. 10.1007/s10519-021-10063-x 34009508

[B116] Zhu S. Wu J. Hu J. (2022). Non-coding RNA in alcohol use disorder by affecting synaptic plasticity. *Exp. Brain Res.* 240 365–379. 10.1007/s00221-022-06305-x 35028694

[B117] Zillich L. Frank J. Streit F. Friske M. M. Foo J. C. Sirignano L. (2021). Epigenome-wide association study of alcohol use disorder in five brain regions. *Neuropsychopharmacology* 47 832–839. 10.1038/s41386-021-01228-7 34775485 PMC8882178

[B118] Zillich L. Poisel E. Frank J. Foo J. C. Friske M. M. Streit F. (2022). Multi-omics signatures of alcohol use disorder in the dorsal and ventral striatum. *Transl. Psychiatry* 12:190. 10.1038/s41398-022-01959-1 35523767 PMC9076849

