# Supplementary material for: Is DNA methylation in the brain a mechanism of alcohol use disorder?
Source: Front Behav Neurosci. 2023 Jan 26;17:957203. doi: 10.3389/fnbeh.2023.957203 (PMC9908583; doi:10.3389/fnbeh.2023.957203)
Supplement: Supplementary file 1 [file Table_1.pdf]

Supplementary Table 1. Differentially methylated genes in the brain after alcohol exposure.

Gene data are based on GeneCards (Stelzer et al., 2016), UniProt (UniProt Consortium, 2021). NAc, nucleus accumbens; m/dPFC, medial/dorsal prefrontal cortex; VS, ventral striatum; VTA, ventral tegmental area; CN, caudate nucleus.

Gene name	Full gene name	Encoded protein function	Species	Brain structure	Experimental model	DNA methylation change	Reference
ARC	*Activity regulated cytoskeleton associated protein*	- predicted to enable mRNA binding activity. Involved in cell migration; cytoskeleton organization; and regulation of cell morphogenesis.	Mouse	amygdala	alcohol training in the IntelliCages	decreased methylation in the promoter and exon 1 correlates with alcohol seeking during withdrawal but not alcohol consumption	(Pagano et al., 2022)
ARHGEF7	*Rho Guanine Nucleotide Exchange Factor 7*	- cytoplasmic protein plays functions in cell migration, attachment and cell spreading as well as promotes the formation of spines and synapses in hippocampal neurons	Rhesus monkey	NAc	alcohol-self administration	methylation levels correlated with average daily alcohol consumption	(Cervera-Juanes et al., 2017)
ARRDC1	*Arrestin Domain Containing 1*	- involved in several processes, including cellular protein metabolic process; extracellular vesicle biogenesis; and negative regulation of Notch signaling pathway	Rat	hypo-thalamus	parents exposed to ethanol	increased methylation levels in promoter region;	(Asimes et al., 2017)
BDNF	*Brain Derived Neurotrophic Factor*	- a member of the nerve growth factor family of proteins, which activates signaling cascades downstream of NTRK2;<br>- during development, it promotes the survival and differentiation of selected neuronal populations of the peripheral and central nervous system. Participates in axonal growth, pathfinding and modulation of dendritic growth and morphology	Rat	NAc, mPFC, amygdala	alcohol-self administration;<br><br>adolescent intermittent ethanol exposure	increased or decreased methylation of promoter region;	(Nieto et al., 2022);<br><br>(Sakharkar et al., 2019)
CACNA1a	*Calcium Voltage-Gated Channel Subunit Alpha 1a*	- mediates the entry of Ca^{2+} ions into excitable cells and are also involved in a variety of calcium-dependent processes, including muscle contraction, hormone or neurotransmitter release, gene expression, cell motility, cell division and cell death	Rat	mPFC	alcohol-dependent animals (chronic intermittent alcohol vapor exposure)	decreased expression; the use of inhibitor of DNA methylation (RG108) prevented the downregulation;	(Barbier et al., 2015)
CACNA1i	*Calcium Voltage-Gated Channel Subunit Alpha 1i*	- mediates the entry of Ca^{2+} ions into excitable cells and are also involved in a variety of calcium-dependent processes, including muscle contraction, hormone or neurotransmitter release, gene expression, cell motility, cell division and cell death	Rat	mPFC	alcohol-dependent animals (chronic intermittent alcohol vapor exposure)	decreased expression	(Barbier et al., 2015)
CDH5	*Cadherin 5*	- calcium-dependent cell adhesion protein, imparting to cells the ability to adhere in a homophilic manner, plays a role in	Rhesus monkey	NAc	alcohol-self administration	methylation levels correlated with average daily alcohol	(Cervera-Juanes et al., 2017)

		endothelial adherens junction assembly and maintenance				consumption	
CPLX2	*Complexin 2*	- cytosolic protein that functions in synaptic vesicle exocytosis. These proteins bind syntaxin, part of the SNAP receptor. The protein product of this gene binds to the SNAP receptor complex and disrupts it, allowing transmitter release<br>- Negatively regulates the formation of synaptic vesicle clustering at the active zone to the presynaptic membrane in postmitotic neurons. Positively regulates a late step in exocytosis of various cytoplasmic vesicles, such as synaptic vesicles and other secretory vesicles	Human	mPFC	AUD patients	differentially methylated promoter regions;	(Wang et al., 2016)
DLGAP2	*DLG Associated Protein 2*	- a membrane-associated protein that may play a role in synapse organization and signaling in neuronal cells	Human	dPFC<br>NAc	AUD patients	differentially methylated region associated with alcohol dependency;	(Meng et al., 2021)
DUSP1	*Dual Specificity Phosphatase 1*	- serine/threonine kinase which acts as an essential component of the MAP kinase signal transduction pathway<br>- plays an important role in the human cellular response to environmental stress as well as in the negative regulation of cellular proliferation	Rat	mPFC	alcohol-self administration (chronic alcohol exposure)	decreased methylation levels of promoter regions and increased mRNA levels;	(Cui et al., 2020)
FAM20B	*Glycosaminoglycan Xylosylkinase*	- responsible for the 2-O-phosphorylation of xylose in the glycosaminoglycan-protein linkage region of proteoglycans thereby regulating the amount of mature GAG chains<br>- predicted to be involved in proteoglycan biosynthetic process	Human	VS	AUD patients	hypermethylated;	(Zillich et al., 2021)
GABRA2	*Gamma-Aminobutyric Acid Type A Receptor Subunit Alpha2*	- GABAA receptors are members of the Cys-loop family of ligand-gated ion channels and, along with GABAB receptors, are responsible for mediating the inhibitory effects of GABA	Human	cerebellum	AUD patients	increased methylation levels in the promoter region, which was correlated with a decrease of mRNA and protein level of *GABRA2* in AUD patients;	(Gatta et al., 2021b)
GABRAD	*Gamma-Aminobutyric Acid Type A Receptor Subunit Delta*	- major inhibitory neurotransmitter in the mammalian brain where it acts at GABA-A receptors, which are ligand-gated chloride channels<br>- mediates neuronal inhibition by binding to the GABA/benzodiazepine receptor and opening an integral chloride channel	Human	cerebellum	AUD patients	increased methylation levels in the promoter region, which was correlated with a decrease of mRNA and protein levels of *GABRD* in AUD patients;	(Gatta et al., 2017)

GDNF	*Glial cell line-derived neurotrophic factor*	- a neurotrophic factor that enhances survival and morphological differentiation of dopaminergic neurons and increases their high-affinity dopamine uptake<br>- May also modulate local neuronal effects in distal regions of the motor neuron	Rat	NAc, VTA	alcohol withdrawal after self-administration	expression and methylation levels of *GDNF* is altered; negative correlation between methylation of negative regulatory element (NRE) of *GDNF* and its mRNA expression;	(Maier et al., 2020)
GPR39	*G Protein-coupled Receptor 39*	- involved in zinc-dependent signaling in epithelial tissue in intestines, prostate and salivary glands as well as in the pathophysiology of depression	Rhesus monkey	NAc	alcohol-self administration	methylation levels correlated with average daily alcohol consumption	(Cervera-Juanes et al., 2017)
GRIN2B	*Glutamate Ionotropic receptor NMDA Type Subunit 2B*	- a subunit of the NMDA receptor ion channel which acts as an agonist binding site for glutamate. The NMDA receptors mediate a slow calcium-permeable component of excitatory synaptic transmission in the central nervous system	Mouse	mPFC	chronic intermittent ethanol exposure	hypomethylation of 18 CpG sites in promoter region and increased expression of mRNA	(Qiang et al., 2014)
H2AC20	*H2A Clustered Histone 20*	- nuclear protein, responsible for the nucleosome structure of the chromosomal fiber in eukaryotes<br>- core component of nucleosome	Human	frontal cortex	AUD patients	increased methylation levels	(Manzardo et al., 2012)
HAP1	*Huntingtin Associated Protein 1*	- neuronal protein that specifically associates with HTT/huntingtin, involved in intracellular trafficking and proposed to link HTT to motor proteins and/or transport cargos<br>- plays a role in vesicular transport within neurons and axons such as from early endosomes to late endocytic compartments and to promote neurite outgrowth	Rat	mPFC	alcohol-self administration (chronic alcohol exposure)	decreased methylation levels of promoter regions and increased mRNA levels;	(Cui et al., 2020)
Hif3a	*Hypoxia-inducible factor 3, alpha subunit*	- the protein encoded by this gene is the alpha-3 subunit of one of several alpha/beta-subunit heterodimeric transcription factors that regulate many adaptive responses to low oxygen tension (hypoxia)	Rat	amygdala	acute alcohol injection	decreased methylation	(Krishnan et al., 2022)
HMGCR	*3-Hydroxy-3-Methylglutaryl-CoA Reductase*	- the rate-limiting enzyme for cholesterol synthesis and is regulated via a negative feedback mechanism mediated by sterols and non-sterol metabolites derived from mevalonate, plays a critical role in cellular cholesterol homeostasis<br>- catalyzes the conversion of (3S)-hydroxy-3-methylglutaryl-CoA (HMG-CoA) to mevalonic acid	Human	CN	AUD patients	hypomethylated;	(Zillich et al., 2021)

IREB2	*Iron Responsive Element Binding Protein 2*	- an RNA-binding protein that acts to regulate iron levels in the cells by regulating the translation and stability of mRNAs that affect iron homeostasis under conditions when iron is depleted	Human	CN	AUD patients	hypomethylated;	(Zillich et al., 2021)
JAKMIP1	*Janus Kinase and Microtubule Interacting Protein 1*	- enables GABA receptor binding activity and RNA binding activity. Involved in cognition	Rhesus monkey	NAc	alcohol-self administration	methylation levels correlated with average daily alcohol consumption	(Cervera-Juanes et al., 2017)
KCNC1	*Potassium Voltage-Gated Channel Subfamily C Member 1*	- voltage-gated potassium channel that plays an important role in the rapid repolarization of fast-firing brain neurons<br>- contributes to fire sustained trains of very brief action potentials at high frequency in pallidal neurons	Rat	mPFC	alcohol-dependent animals (chronic intermittent alcohol vapor exposure)	decreased expression;	(Barbier et al., 2015)
KIRREL3	*Kirre like Nephrin Family Adhesion Molecule 3*	- synaptic adhesion molecule required for the formation of target-specific synapses	Rhesus monkey	NAc	alcohol-self administration	methylation levels correlated with average daily alcohol consumption	(Cervera-Juanes et al., 2017)
LRP5	*Low-density Lipoprotein Receptor-related Protein 5*	- low-density lipoprotein receptor that binds and internalizes ligands in the process of receptor-mediated endocytosis	Rhesus monkey	NAc	alcohol-self administration	methylation levels correlated with average daily alcohol consumption	(Cervera-Juanes et al., 2017)
NBEA	*Neurobeachin*	- Binds to type II regulatory subunits of protein kinase A and targets them to the membrane	Rhesus monkey	NAc	alcohol-self administration	methylation levels correlated with average daily alcohol consumption	(Cervera-Juanes et al., 2017)
NPY	*Neuropeptide Y*	- a neuropeptide that is widely expressed in the central nervous system and influences many physiological processes, including cortical excitability, stress response, food intake, circadian rhythms, and cardiovascular function	Rat	amygdala	alcohol-self administration (adolescent intermittent ethanol exposure)	increased levels of DNA methylation in the promoter region;	(Sakharkar et al., 2019)
NR3C1	*Nuclear Receptor Subfamily 3 Group C Member 1*	- glucocorticoid receptor, which can function both as a transcription factor that binds to glucocorticoid response elements in the promoters of glucocorticoid responsive genes to activate their transcription, and as a regulator of other transcription factors<br>- involved in inflammatory responses, cellular proliferation, and differentiation in target tissues	Human	PFC	AUD patients	increased methylation of the NR3C1 exon variant 1H, which were associated with reduced levels of mRNA and protein;	(Gatta et al., 2021a)

NTF3	*Neurotrophin 3*	- a member of the neurotrophin family that controls survival and differentiation of mammalian neurons (visceral and proprioceptive sensory neurons)<br>- Involved in the maintenance of the adult nervous system, and may affect development of neurons in the embryo when it is expressed in the human placenta.	Rat	mPFC	alcohol-self administration (chronic alcohol exposure)	increased methylation levels of promoter regions and decreased mRNA levels in alcohol-exposed animals	(Cui et al., 2020)
			Rat	mPFC	alcohol-self administration (chronic alcohol exposure)	downregulation of *NTF3* expression, which was reversed after the use of methylation inhibitor (5'AZA)	(Qiao et al., 2017)
NTM	*Neurotrimin*	- neural cell adhesion molecule promotes neurite outgrowth and adhesion via a homophilic mechanism	Rhesus monkey	NAc	alcohol-self administration	methylation levels correlated with average daily alcohol consumption	(Cervera-Juanes et al., 2017)
NTRK3	*Neurotrophic Receptor Tyrosine Kinase 3*	-a receptor tyrosine kinase involved in nervous system and probably heart development<br>- Upon binding of its ligand NTF3, autophosphorylates and activates different signaling pathways, including the phosphatidylinositol 3-kinase/AKT and the MAPK pathways, that control cell survival and differentiation	Rat	mPFC	alcohol-self administration (chronic alcohol exposure)	no differences in the expression, however decreased expression after methylation inhibition (the use of 5'AZA)	(Qiao et al., 2017)
OPRK1	*Opioid Receptor Kappa 1*	- an opioid receptor plays a role in the perception of pain and mediating the hypolocomotion, analgesic and aversive actions of synthetic opioids	Rat	NAc	alcohol-self administration in alcohol preferring rats	no differences in 5mC and increased levels of 5hmC in the promoter region; increased mRNA levels;	(Niinep et al., 2021)
PCAT29	*Prostate Cancer Associated Transcript 29*	- gene is thought to produce a functional long non-coding RNA. This transcript was identified in prostate cancer cells and may suppress tumor formation	Human	VS	AUD patients	hypermethylated;	(Zillich et al., 2021)
PDYN	*Prodynorphin*	- plays a role in a number of physiologic functions, including pain perception and responses to stress	Rat	NAc	alcohol-self administration in alcohol preferring rats	decreased levels of 5mC in the promoter region and increased mRNA levels	(Niinep et al., 2021)
PDYN	*Prodynorphin*	- play a role in a number of physiologic functions, including pain perception and responses to stress	Human	mPFC	AUD patients	differentially methylated CpG-SNP associated with alcoholism;	(Taqi et al., 2011)
PEG10	*Paternally Expressed 10*	- forms virion-like extracellular vesicles that encapsulate their own mRNA and are released from cells, enabling intercellular transfer of PEG10 mRNA	Human	mPFC	AUD patients	differentially methylated promoter regions;	(Wang et al., 2016)

PPM1G	*Protein Phosphatase Magnesium-dependent 1 Gamma*	- a member of the PP2C family of Ser/Thr protein phosphatases, known to be negative regulators of cell stress response pathways<br>- responsible for the dephosphorylation of Pre-mRNA splicing factors, which is important for the formation of a functional spliceosome	Rat	mPFC	alcohol-self administration (chronic alcohol exposure)	increased methylation levels of promoter regions and decreased mRNA levels;	(Cui et al., 2020)
Slc10a6	*Solute Carrier Family 10 Member 6*	- predicted to be involved in bile acid and bile salt transport.	Rat	amygdala	acute alcohol injection	decreased methylation	(Krishnan et al., 2022)
SLC30A8	*Solute Carrier Family 30 Member 8*	- zinc efflux transporter involved in the accumulation of zinc in intracellular vesicles<br>- may be a major component for providing zinc to insulin maturation and/or storage processes in insulin-secreting pancreatic beta-cell	Human	VS	AUD patients	hypermethylated;	(Zillich et al., 2021)
SNORD42A	*U42A small nuclear RNA*	- an RNA Gene, and is affiliated with the snoRNA class	Human	mPFC	AUD patients	differentially methylated promoter regions;	(Wang et al., 2016)
SYT1	*Synaptotagmin 1*	- calcium sensor that participates in triggering neurotransmitter release at the synapse<br>- integral membrane protein of synaptic vesicles thought to serve as Ca^{2+} sensors in the process of vesicular trafficking and exocytosis	Rat	mPFC	alcohol-dependent animals (chronic intermittent alcohol vapor exposure)	decreased mRNA expression; the use of inhibitor of DNA methylation (RG108) prevented the downregulation;	(Barbier et al., 2015)
SYT2	*Synaptotagmin 2*	- a synaptic vesicle membrane protein, which functions as a Ca^{2+} sensor in vesicular trafficking and exocytosis<br>- may have a regulatory role in the membrane interactions during trafficking of synaptic vesicles at the active zone of the synapse	Rat	mPFC	alcohol-dependent animals (chronic intermittent alcohol vapor exposure)	decreased expression; the use of inhibitor of DNA methylation (RG108) prevented the downregulation as well as alcohol-induced hypermethylation on the first exon;	(Barbier et al., 2015)
WNK1	*WNK Lysine Deficient Protein Kinase 1*	- a member of the WNK subfamily of serine/threonine protein kinases<br>- plays an important role in the regulation of electrolyte homeostasis, cell signaling, survival and proliferation. Acts as an activator and inhibitor of sodium-coupled chloride cotransporters and potassium-coupled chloride cotransporters respectively	Rat	mPFC	alcohol-dependent animals (chronic intermittent alcohol vapor exposure)	decreased expression	(Barbier et al., 2015)

WNK2	*WNK Lysine Deficient Protein Kinase 2*	- a cytoplasmic serine-threonine kinase that belongs to the protein kinase superfamily<br>- plays an important role in the regulation of electrolyte homeostasis, cell signaling survival, and proliferation	Rat	mPFC	alcohol-dependent animals (chronic intermittent alcohol vapor exposure)	decreased expression;<br>the use of inhibitor of DNA methylation (RG108) prevented the downregulation;	(Barbier et al., 2015)
ZFHX3	*Zinc Finger Homeobox 3*	- transcription factor with multiple homeodomains and zinc finger motifs, and regulates myogenic and neuronal differentiation<br>- may act as an activator or a repressor. Inhibits the enhancer element of the AFP gene by binding to its AT-rich core sequence. In concert with SMAD-dependent TGF-beta signaling can repress the transcription of AFP via its interaction with SMAD2/3	Human	mPFC	AUD patients	differentially methylated promoter regions;	(Wang et al., 2016)

Asimes, A., Torcaso, A., Pinceti, E., Kim, C. K., Zeleznik-Le, N. J., and Pak, T. R. (2017). Adolescent binge-pattern alcohol exposure alters genome-wide DNA methylation patterns in the hypothalamus of alcohol-naïve male offspring. *Alcohol Fayettev. N* 60, 179–189. doi: 10.1016/j.alcohol.2016.10.010.

Barbier, E., Tapocik, J. D., Juergens, N., Pitcairn, C., Borich, A., Schank, J. R., et al. (2015). DNA methylation in the medial prefrontal cortex regulates alcohol-induced behavior and plasticity. *J. Neurosci. Off. J. Soc. Neurosci.* 35, 6153–6164. doi: 10.1523/JNEUROSCI.4571-14.2015.

Cervera-Juanes, R., Wilhelm, L. J., Park, B., Grant, K. A., and Ferguson, B. (2017). Alcohol-dose-dependent DNA methylation and expression in the nucleus accumbens identifies coordinated regulation of synaptic genes. *Transl. Psychiatry* 7, e994–e994. doi: 10.1038/tp.2016.266.

Cui, H. Z., Sun, M. Z., Wang, R. Z., Li, C. Y., Huang, Y. X., Huang, Q. J., et al. (2020). DNA methylation in the medial prefrontal cortex regulates alcohol-related behavior in rats. *Yi Chuan Hered.* 42, 112–125. doi: 10.16288/j.yczz.19-261.

Gatta, E., Auta, J., Gavin, D. P., Bhaumik, D. K., Grayson, D. R., Pandey, S. C., et al. (2017). Emerging Role of One-Carbon Metabolism and DNA Methylation Enrichment on δ-Containing GABAA Receptor Expression in the Cerebellum of Subjects with Alcohol Use Disorders (AUD). *Int. J. Neuropsychopharmacol.* 20, 1013–1026. doi: 10.1093/ijnp/pyx075.

Gatta, E., Grayson, D. R., Auta, J., Saudagar, V., Dong, E., Chen, Y., et al. (2021a). Genome-wide methylation in alcohol use disorder subjects: implications for an epigenetic regulation of the cortico-limbic glucocorticoid receptors (NR3C1). *Mol. Psychiatry* 26, 1029–1041. doi: 10.1038/s41380-019-0449-6.

Gatta, E., Guidotti, A., Saudagar, V., Grayson, D. R., Aspesi, D., Pandey, S. C., et al. (2021b). Epigenetic Regulation of GABAergic Neurotransmission and Neurosteroid Biosynthesis in Alcohol Use Disorder. *Int. J. Neuropsychopharmacol.* 24, 130–141. doi: 10.1093/ijnp/pyaa073.

Maier, H. B., Neyazi, M., Neyazi, A., Hillemacher, T., Pathak, H., Rhein, M., et al. (2020). Alcohol consumption alters Gdnf promoter methylation and expression in rats. *J. Psychiatr. Res.* 121, 1–9. doi: 10.1016/j.jpsychires.2019.10.020.

Manzardo, A. M., Henkhaus, R. S., and Butler, M. G. (2012). Global DNA promoter methylation in frontal cortex of alcoholics and controls. *Gene* 498, 5–12. doi: 10.1016/j.gene.2012.01.096.

Meng, W., Sjöholm, L. K., Kononenko, O., Tay, N., Zhang, D., Sarkisyan, D., et al. (2021). Genotype-dependent epigenetic regulation of DLGAP2 in alcohol use and dependence. *Mol. Psychiatry* 26, 4367–4382. doi: 10.1038/s41380-019-0588-9.

Nieto, S. J., Haile, C. N., Quave, C. B., Harding, M. J., Nielsen, D. A., Meisch, R. A., et al. (2022). Paternal alcohol exposure reduces acquisition of operant alcohol self-administration and affects Bdnf DNA methylation in male and female offspring. *Addict. Biol.* 27, e13078. doi: 10.1111/adb.13078.

Niinep, K., Anier, K., Eteläinen, T., Piepponen, P., and Kalda, A. (2021). Repeated Ethanol Exposure Alters DNA Methylation Status and Dynorphin/Kappa-Opioid Receptor Expression in Nucleus Accumbens of Alcohol-Preferring AA Rats. *Front. Genet.* 12. Available at: https://www.frontiersin.org/article/10.3389/fgene.2021.750142 [Accessed January 19, 2022].

Pagano, R., Salamian, A., Zielinski, J., Beroun, A., Nalberczak-Skóra, M., Skonieczna, E., et al. (2022). Arc controls alcohol cue relapse by a central amygdala mechanism. *Mol. Psychiatry*. doi: 10.1038/s41380-022-01849-4.

Qiang, M., Li, J. G., Denny, A. D., Yao, J.-M., Lieu, M., Zhang, K., et al. (2014). Epigenetic mechanisms are involved in the regulation of ethanol consumption in mice. *Int. J. Neuropsychopharmacol.* 18, pyu072. doi: 10.1093/ijnp/pyu072.

Qiao, X., Yin, F., Ji, Y., Li, Y., Yan, P., and Lai, J. (2017). 5-Aza-2'-deoxycytidine in the medial prefrontal cortex regulates alcohol-related behavior and Ntf3-TrkC expression in rats. *PloS One* 12, e0179469. doi: 10.1371/journal.pone.0179469.

Sakharkar, A. J., Kyzar, E. J., Gavin, D. P., Zhang, H., Chen, Y., Krishnan, H. R., et al. (2019). Altered amygdala DNA methylation mechanisms after adolescent alcohol exposure contribute to adult anxiety and alcohol drinking. *Neuropharmacology* 157, 107679. doi: 10.1016/j.neuropharm.2019.107679.

Stelzer, G., Rosen, N., Plaschkes, I., Zimmerman, S., Twik, M., Fishilevich, S., et al. (2016). The GeneCards Suite: From Gene Data Mining to Disease Genome Sequence Analyses. *Curr. Protoc. Bioinforma.* 54, 1.30.1-1.30.33. doi: 10.1002/cpbi.5.

Taqi, M. M., Bazov, I., Watanabe, H., Sheedy, D., Harper, C., Alkass, K., et al. (2011). Prodynorphin CpG-SNPs associated with alcohol dependence: elevated methylation in the brain of human alcoholics. *Addict. Biol.* 16, 499–509. doi: 10.1111/j.1369-1600.2011.00323.x.

UniProt Consortium (2021). UniProt: the universal protein knowledgebase in 2021. *Nucleic Acids Res.* 49, D480–D489. doi: 10.1093/nar/gkaa1100.

Wang, F., Xu, H., Zhao, H., Gelernter, J., and Zhang, H. (2016). DNA co-methylation modules in postmortem prefrontal cortex tissues of European Australians with alcohol use disorders. *Sci. Rep.* 6, 19430. doi: 10.1038/srep19430.

Zillich, L., Frank, J., Streit, F., Friske, M. M., Foo, J. C., Sirignano, L., et al. (2021). Epigenome-wide association study of alcohol use disorder in five brain regions. *Neuropsychopharmacology*, 1–8. doi: 10.1038/s41386-021-01228-7.
